# Clinical strains isolated from early-stage colorectal cancer patients promote tumorigenesis

**DOI:** 10.7717/peerj.21488

**Published:** 2026-07-14

**Authors:** Daisuke Suzuki, Jiayue Yang, Nozomu Obana, Shinichi Yachida, Satoshi Shiba, Sayaka Mizutani, Hiroyuki Takamaru, Yutaka Saito, Shinji Fukuda, Takuji Yamada

**Affiliations:** 1 School of Life Science and Technology, Institute of Science Tokyo, Tokyo, Japan; 2 Institute for Advanced Biosciences, Keio University, Yamagata, Japan; 3 Transborder Medical Research Center, University of Tsukuba, Ibaraki, Japan; 4 Microbiology Research Center for Sustainability, University of Tsukuba, Ibaraki, Japan; 5 Department of Cancer Genome Informatics, The University of Osaka, Osaka, Japan; 6 Japan Agency for Medical Research and Development—Core Research for Evolutional Science and Technology (AMED–CREST), Japan Agency for Medical Research and Development, Tokyo, Japan; 7 Integrated Frontier Research for Medical Science Division, Institute for Open and Transdisciplinary Research Initiatives (OTRI), The University of Osaka, Osaka, Japan; 8 Division of Cancer Genomics, National Cancer Center Research Institute, Tokyo, Japan; 9 Endoscopy Division, National Cancer Center Hospital, Tokyo, Japan; 10 Gut Environmental Design Group, Kanagawa Institute of Industrial Science and Technology, Kanagawa, Japan; 11 Innovative Microbiome Therapy Research Center Laboratory for Regenerative Microbiology, Juntendo University, Tokyo, Japan; 12 Metagen, Inc., Tsuruoka, Yamagata, Japan; 13 Metagen Therapeutics, Inc., Tsuruoka, Yamagata, Japan; 14 digzyme, Inc., Minato-ku, Tokyo, Japan

**Keywords:** Strain diversity, Early-stage colorectal cancer, Comparative genomics, Gut microbiota

## Abstract

**Background:**

Colorectal cancer (CRC) is prevalent worldwide and is associated with gut commensals. Recent studies have highlighted the effects of gut microbes on CRC development driven by their strain diversity. Nevertheless, the impact of the gut microbial community on tumorigenesis in early-stage (ES) CRC remains unexplored.

**Methods:**

To assess the potential gut microbial community, which is critical to tumorigenesis in early-stage CRC, we collected publicly available shotgun metagenomes from CRC patient faecal samples from a Japanese population. Correlation analysis of the microbial profiles derived from the metagenomes revealed an ES CRC-associated community. To elucidate the strain diversity of the targeted community, we isolated strains from ES CRC patient faecal samples and employed comparative genomics. To evaluate the strain-specific effects of the community on tumorigenesis, we introduced an isolated strain cocktail into a CRC mouse model.

**Results:**

Among the most significant ES CRC-associated species, we identified* Lancefieldella parvula* (Lp), as reported in a previous study. The 20 species were identified as positively correlated with Lp. Seven of the 20 species were associated with ES CRC, including *Actinomyces* and *Solobacterium*. *Schaalia odontolytica* (So) (formerly known as *Actinomyces odontolyticus)* and *Solobacterium moorei* (Sm) were previously reported as potential species that promote CRC. Thus, we isolated clinical strains of Lp, So, and Sm from faecal samples as potential members of the ES CRC-associated community. Comparative genomics revealed that iron-related genes were shared among clinical strains. In the oral challenge with clinical strains, namely, Lp, So, and Sm, the mice exhibited shorter survival and significantly increased tumorigenesis, suggesting that the cocktail of clinical strains is more pathogenic to the CRC mouse model than the type strain is. In summary, we inferred that the ES CRC-associated community could promote CRC, and the effects depend on the strains involved.

## Introduction

Colorectal cancer (CRC) is the third most common cancer worldwide (Sung et al., 2021) and needs to be addressed with public health measures. Most cases are attributable to sporadic CRC, which is not associated with genetic predisposition or family history (Burt, 2000), and its onset is related to lifestyle factors. Lifestyle factors are known to alter our gut commensals, and the gut microbial profiles are dependent on CRC status and aligned with the adenoma–carcinoma sequence (Feng et al., 2015; Thomas et al., 2019; Yachida et al., 2019; Zeller et al., 2014). A recent study highlighted the causative effects of specific gut microbes on tumorigenesis *in vitro* and *in vivo* (Chen et al., 2025; Long et al., 2019; Miyakawa et al., 2024; Rubinstein et al., 2013), and CRC-associated bacteria are recognized as potential therapeutic targets (Brennan et al., 2021). A large meta-analysis highlighted the importance of functional heterogeneity among CRC-associated species spanning diverse taxa (Piccinno et al., 2025).

Despite the maturity of previous studies on CRC and gut microbes, few studies have focused on the initiation or transition from normal or adenoma cases to early-stage (ES) CRC. To understand the association between CRC and gut microbes, assessing the microbial signature at the carcinogenesis step *in vivo* is essential. Additionally, few studies have investigated the effects of the microbial community on CRC, while a machine learning model identified four distinct communities among CRC patients across several populations (Rynazal et al., 2023). To understand the effects of the gut microbial community on ES CRC, we aimed to (1) infer the early-stage CRC-associated community, (2) isolate clinical strains and understand gene functional diversity compared with that of type strains, and (3) evaluate the effect of ES CRC-associated strains on tumorigenesis in a CRC mouse model.

## Materials & Methods

### Ethics approval and consent to participate

The faecal samples and isolated strains used in this study were obtained with written informed consent for all participants and with the approval of the institutional review boards of each participating institute (National Cancer Center, 2013–244; Institute of Science Tokyo, 2014018). Mouse experiments were carried out in a humane manner after receiving approval from the Institutional Animal Care and Use Committee of the University of Tsukuba (24-44) and in accordance with the Regulations for Animal Experiments of our university and the Fundamental Guidelines for Proper Conducting of Animal Experiments and Related Activities in Academic Research Institutions under the jurisdiction of the Ministry of Education, Culture, Sports, Science and Technology.

### Shotgun metagenome collections and taxonomic profiling

The shotgun metagenomes used in this study were collected from a publicly available dataset previously sequenced in Japan (Yachida et al., 2019). The metagenomes of recruited patients analyzed in this study were confirmed to be free of a hereditary or suspected hereditary disease, inflammatory bowel disease and surgery (Yachida et al., 2019). The sequence read data from JPN were obtained from the DDBJ Sequence Read Archive under the study identifier PRJDB4176. The terminology related to the columnar epithelium-lined gastrointestinal (GI) tract was previously reported (Djinbachian et al., 2024). The obtained metagenomes were profiled by MetaPhlAn4, a method for detecting unique clade-specific marker genes arranged by species-level genome bins (SGBs) (Blanco-Míguez et al., 2023; Truong et al., 2017).

To validate the ES CRC-associated species, publicly available 16S rRNA gene amplicon sequencing from three external cohorts (denoted as Cohort 1, Cohort 2 and Cohort 3) of two studies recruited in Japan were collected (Konishi et al., 2022; Okumura et al., 2021). To profile the phylogenetic composition, quality control and subsequent analysis of 16S rRNA gene sequence were performed *via* QIIME2 (version 2024.10.1) (Bolyen et al., 2019). The amplicon sequencing data were imported with tools import –type ‘SampleData[PairedEndSequencesWithQuality]’ –input-format CasavaOneEightSingleLanePerSampleDirFmt options. For Cohort 1 and 2, the sequence reads were trimmed and truncated with –p-trim-left-f 20 –p-trim-left-r 20 –p-trunc-len-f 240 –p-trunc-len-r 240 options. For cohort 3, the demultiplexed sequence reads were trimmed and truncated with –p-trim-left-f 20 –p-trim-left-r 20 –p-trunc-len-f 220 –p-trunc-len-r 160 options. The taxonomy of representative sequences was assigned with 16S rRNA gene sequence reference (DDBJ reference 130.0, Apr. 2023) *via* BLASTN (version 2.14.0) (Camacho et al., 2009) with -outfmt 6 -perc_identity 95 option. The genes with alignment identity over 98.65, and coverage over 0.99 were screened, and the gene whose identity was maximal was used as the reliably annotated 16S rRNA gene.

### Early-stage CRC-associated species and communities

The relative abundance profiles of the normal and Stage 0 or multiple polyps (as precancerous lesions) populations were summarized and tested with the Mann–Whitney U test. The results were adjusted to control the false discovery rate with false_discovery_control provided by SciPy (Virtanen et al., 2020), and the species (FDR < 0.1) were considered associated with ES CRC. The prevalence of each species was estimated by counting the number of samples that satisfied the criterion of having an abundance greater than zero. To validate robustness of the detected ES CRC-associated species, the multivariable associations which incorporated potential confounders such as age, gender or body mass index (BMI) were conducted *via* MaAsLin3 (Nickols et al., 2026) with formula=‘∼Age + BMI + Group + Gender’ after centered log-ratio normalisation and set reference as Normal. We used Fastspar (Watts et al., 2019) to conduct a correlation analysis of the “estimated_number_of_reads_from_the_clade” value in MetaPhlAn4 profiles. The correlation values were screened according to the criteria requiring both correlation coefficient > 0.5 and *p* < 0.01. For network illustration, yEd was used for the overall network with an organic layout, and for the partial network involving *Lancefieldella parvula* (Lp)*,* a radial layout was used.

### Strain isolation from the faecal samples

To isolate clinical strains, patients were selected on the basis of the Lp, *Schaalia odontolytica* (So) and *Solobacterium moorei* (Sm) relative abundance in faecal samples from the Japanese population. Frozen faecal samples were suspended in GAM broth (Shimadzu Diagnostics Corporation, Kyoto, Japan) at 10 mg/ml and spread onto a GAM agar plate supplemented with a combination of antibiotics (Table S1). After incubation for four days at 37 °C in an anaerobic chamber (COY), emerging colonies were picked and inoculated into 200 µL of GAM broth in 96-well plates. We used an *Atopobium cluster* primer pair (c-Atopo-F, 5′-GGGTTGAGAGACCGACC-3′; c-Atopo-R, 5′-CGGRGCTTCTTCTGCAGG-3′) (Matsuki et al., 2004), a *Schaalia odontolytica*-specific primer pair (Sodo_F, 5′-GCCACCCGTGGTTTCTGCG-3′; Sodo_R, 5′-GCACAAAGCGGTTAGGCCAT-3′) (Kobayashi et al., 2018) and a *Solobacterium moorei*-specific primer pair (SomF, 5′-TCGGAAGGCATCTTCTGGTT-3′; SomR, 5′-AAGTGGCTGGATTGGGTTGA-3′) (Furuichi, Fuchigami & Tsuzukibashi, 2020) for diagnostic PCR. PCR-positive cultures were restreaked onto GAM plates, and a single colony of each strain was isolated. In this study, we obtained clinical isolates for Lp (Lp_C), So (So_C), and Sm (Sm_C). The 16S rRNA genes of each isolated strain were identified *via* Sanger sequencing. The type strains of *L. parvula* (JCM 10300, Lp_T), *S. odontolytica* (JCM 14871, So_T), and *S. moorei* (JCM 10645, Sm_T) were purchased from the Japanese Collection of Microorganisms (JCM) for comparison with the clinical strains.

### Sequences of isolated strain genomes and assemblies

Genomic DNA was extracted from the isolated strains grown on GAM plates supplemented with 5% defibrinated horse blood for 2 days using a Monarch^®^ Genomic DNA Purification Kit (New England Biolabs, Ipswich, MA, USA) according to the manufacturer’s instructions. Whole-genome shotgun sequencing was carried out on the PacBio Sequel IIe platform, which was used to sequence the Lp_C, Sm_C, So_C, and So_T strains. The circular consensus sequences from the PacBio Sequel II platform were used for assembly by Flye (Kolmogorov et al., 2019). The housekeeping gene *dnaA* of the assembled genome was detected by Prokka (Seemann, 2014), and the genome sequence was rotated and flipped so that dnaA was located at the beginning of the forward strand following Unicycer methods (Wick et al., 2017). The complete genomes of Lp_T were obtained from the NCBI RefSeq assembly https://www.ncbi.nlm.nih.gov/datasets/genome/GCF_000024225.1/, and the complete genomes of Sm_T were obtained from DDBJ AP028934 (Oshibuchi et al., 2024). FastANI (Jain et al., 2018) was used to calculate the average nucleotide identity between the type and clinical strains. Genome quality was assessed using CheckM (Parks et al., 2015). Their taxonomy was determined using 16S rRNA genes (DDBJ reference 130.0, Apr. 2023) and unique clade-specific marker genes with the MetaPhlAn4 database using BLASTN (Camacho et al., 2009).

### Comparative genomics

The gene profiles of the genomes were annotated with Prokka (Seemann, 2014) and screened with Pseudofinder (Syberg-Olsen et al., 2022) to eliminate potential pseudogene effects. The intact-gene-derived faa files were subsequently annotated by the Virulence Factor Database (VFDB) (Liu et al., 2022) with DIAMOND (Buchfink, Reuter & Drost, 2021). Genes with a bitscore > 100 and coverage > 0.8 were used, and the gene with the highest bitscore was designated as a reliably annotated gene. The intact-gene-derived GFF files were analyzed by Panaroo (Tonkin-Hill et al., 2020), and pangenomes of each species were constructed. The gene-unannotated orthologues were not considered in further analysis. The pathways of the genomes were annotated by gapseq (Zimmermann, Kaleta & Waschina, 2021). The gene, virulence factor, and pathway profiles were compared between the type and clinical strains of each species. To confirm the CnaB-type domain-containing protein, in addition to Prokka and VFDB-annotated results, we used the Pfam-A database (https://ftp.ebi.ac.uk/pub/databases/Pfam/current_release/) provided by EMBL-EBI with HMMER (Eddy, 2011) to confirm the presence of the CnaB-type domain-containing protein (PF05738.20). To elucidate the distribution of CnaB-type domain-harbouring species, complete and reference genomes corresponding to the abovementioned species were obtained from NCBI Refseq. To avoid contradictions in the nomenclature of species, we employed the List of Prokaryotic names with Standing in Nomenclature (LPSN) (Parte et al., 2020). The curated genomes were annotated by Prokka, and the protein sequence files were searched by HMMER.

### Mouse experiments

Humane endpoint criteria were established prior to the experiment and included >20% body weight loss. No animals required euthanasia prior to the planned experimental endpoint. All the mice (C57BL/6-ApcMin^/+^, *N* = 29) were caged in the specific pathogen free (SPF) condition, and obtained by mating ApcMin^/+^ male mice (purchased from the Jackson Laboratory) with wild-type female mice and were genotyped by PCR using the APC region. All the mice were maintained at a temperature of 23.5 °C (±2.5 ° C) and a humidity of 52.5% (±12.5%) under 200 lx illumination with a light:dark cycle of 14:10 h. The mice were housed five per cage on standard bedding and provided ad libitum with drinking water and a standard chow, MF diet (Oriental Yeast Co., Ltd.). Six-week-old mice were acclimatized to AIN-93G (Oriental Yeast, Co. Ltd.). The drinking water of seven-week-old mice was replaced with water containing 1.0 g/L ampicillin, 1.0 g/L neomycin, and 0.5 g/L vancomycin for the depletion of gut bacteria. Eight-week-old mice were divided into four groups: those inoculated with sterilized GAM (control), the type-strain cocktail (Type_3Mix; Lp_T, So_T, and Sm_T), the type-clinical-strain cocktail (Clinical_3Mix; Lp_C, So_C, and Sm_C), and *F. nucleatum* (positive control).

The bacterial suspension (10^8^ CFU) was orally administered to the mouse gut 3 times/week until 13 weeks of age. Because this intervention was non-invasive, no analgesia was applied. To confirm that there were no significant differences in mouse body weights among groups, a Kruskal–Wallis test was performed using SciPy (Virtanen et al., 2020).

After the mice were anaesthetized with isoflurane (FUJIFILM Wako Pure Chemical Corporation) at 4% for induction, and cardiac blood was collected as a terminal procedure. This procedure was followed by euthanasia by cervical dislocation, and subsequent resection of the small and large intestines. The tissue was rinsed with sterilized PBS and immersed in 10% neutral formalin (pH 7.4) buffer solution (FUJIFILM Wako Pure Chemical Corporation). Primary outcomes measured in this study were (1) the survival rate, and (2) the formed polyp numbers of survived mice counted by the same person, followed by the analysis conducted by another person. To confirm the survival rate differences in between control and test groups, a log-rank test was performed using lifelines (Davidson-Pilon, 2019). To confirm the promoted tumorigenesis effects of bacterial inoculation, a Mann–Whitney U test was employed using SciPy (Virtanen et al., 2020). All surviving mice were euthanized and subsequently analyzed. To minimize the effects of confounders, the above procedures were carried out by dividing the mice into three groups.

## Results

### Detection of early-stage CRC-associated species

To detect early-stage (ES) CRC-associated species, publicly available metagenomes of CRC patient faecal samples were characterized using MetaPhlAn4, a tool for profiling the gut microbiome (Blanco-Míguez et al., 2023). The metagenomes (*N* = 574) covered normal (*N* = 251, without carcinoma), multiple polyps (*N* = 66), Stage 0 CRC (*N* = 73, as ES), Stage I/II  CRC (*N* = 110), and Stage III/IV CRC (*N* = 74 as late-stage) samples of the Japanese population (Yachida et al., 2019), and the microbial profiles from Stage 0 patients were compared with those of normal individuals to elucidate the carcinogenic step in the adenoma–carcinoma sequence. Stage 0 CRC (carcinoma *in situ*) corresponds to ES CRC according to the Japanese classification, while it is classified as high-grade dysplasia in the World Health Organization and American Joint Committee on Cancer criteria (Djinbachian et al., 2024).

**Figure 1:**
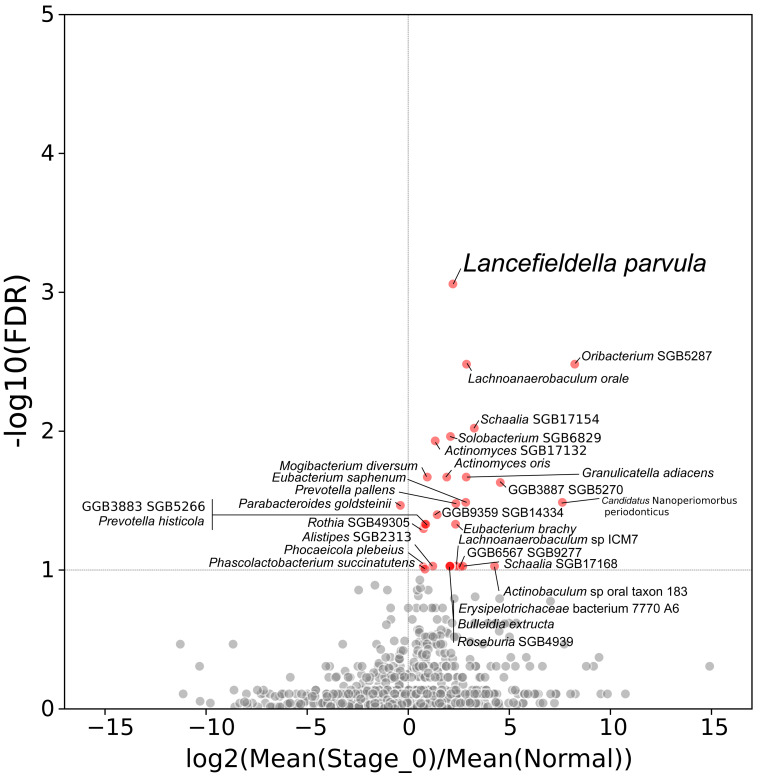
Enriched and depleted species in early-stage CRC relative to normal. Red: significantly associated species (FDR < 0.1); grey: not significant.

**Figure 2:**
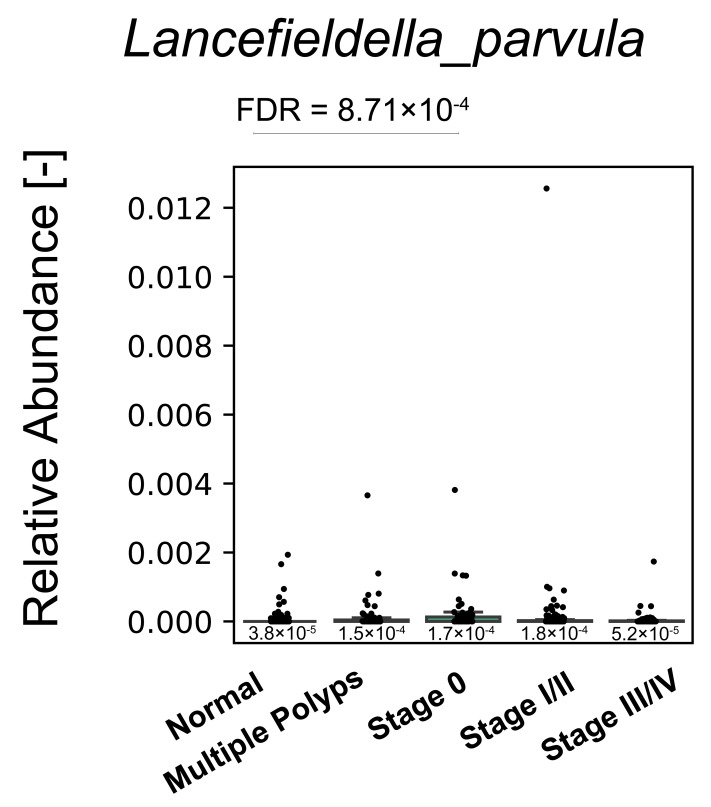
Relative abundance of *Lancefieldella parvula* (Lp) through the adenoma-carcinoma sequence. It was enriched in Stage 0 compared with the normal individuals and gradually decreased in later stages. The mean abundance values for each stage are shown below the boxplots.

After testing the relative abundance with the Mann–Whitney U test followed by the Benjamini–Hochberg procedure, we observed 29 species associated with Stage 0 compared with normal (FDR < 0.1) (Fig. 1, Table S2). One species, including *Parabacteroides goldsteinii* (FDR = 0.0342), were depleted in Stage 0, while 28 species were enriched. Lp was the most significantly enriched species in Stage 0 (FDR = 8.71 ×10^−4^) and was consistently detected in the original study by qPCR (Yachida et al., 2019). Lp was reported as a potential pancolitis inducer and mediator of H_2_S producers (Mottawea et al., 2016). This species was enriched in the Stage 0 population but less enriched in later stages; thus, it was assumed to be an ES CRC-associated species (Fig. 2). To ascertain the robustness of the results with Lp, MaAsLin3, the computational tool for analyzing multivariable associations used to consider the effects of confounders such as age, gender or BMI (Nickols et al., 2026). Although age, gender or BMI were associated with various taxa, the significant enrichment of Lp in Stage 0 was confirmed by both abundance and prevalence, independent of associations with other factors (Table S3). Moreover, Lp was significantly enriched in precancerous stage (multiple polyps) (FDR = 9.49 ×10^−2^), suggesting its potential involvement prior to carcinogenesis (Fig. S2, Table S4).

**Figure 3:**
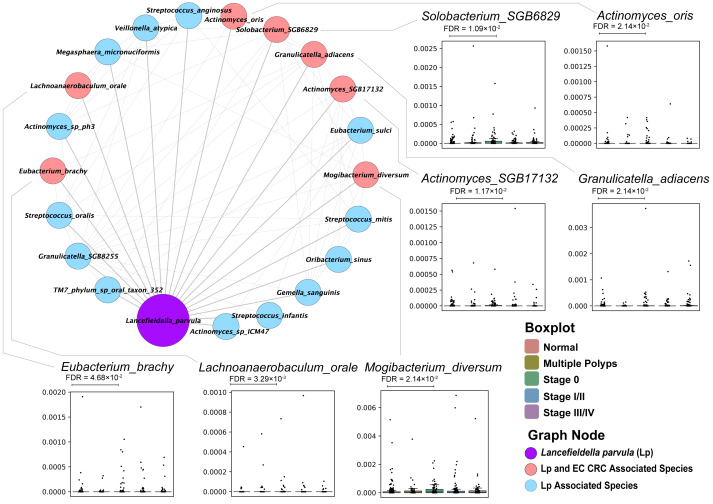
Lp-involving community in early-stage CRC inferred by the positive correlation of the relative abundance. The network edge shows a significant positive correlation between the two species (*p* < 0.01; Fastspar correlation value > 0.5). Red nodes: Lp-associated and early-stage CRC-associated species; blue nodes: Lp-associated species. The boxplots were presented for the species represented by the red nodes. The overview of the community was illustrated in Fig. S3.

To assess the ES CRC-associated bacterial community, we employed FastSpar, a tool that robustly infers abundance correlations from compositional data (Watts et al., 2019). On the basis of the valid edges (*p* < 0.01, correlation value > 0.5), correlation networks were constructed to infer the ES CRC-associated community. Although four clusters were observed in the normal population, we observed five clusters (>10 nodes) in Stage 0, and cluster 2 was composed of 17 ES CRC-associated species, including Lp (Fig. S3). The ES CRC-associated species tended to have a greater number of edges (degree) (*p* = 0.00367), suggesting their importance in the ES CRC-associated microbial community (Fig. S4). To understand the community involving Lp, we focused on Lp and its adjacent nodes (Fig. 3). The Lp-associated species overlapped with seven ES-associated species out of 20 positively correlated species, including *Actinomyces* and *Solobacterium*. The correlation values with Lp were 0.56 and 0.66, and the prevalence in Stage 0/Normal was 0.29/0.10 and 0.44/0.22 for *Actinomyces* SGB17132 and *Solobacterium* SGB6829, respectively, indicating their close relationship with Lp and enrichment in Stage 0. In addition to relative abundance, although the prevalence tended to increase in Stage 0, the proportion of ES CRC-associated species-positive individuals remained below 50% (Fig. S5A), suggesting the substantial heterogeneity within the population, even within a single cohort study and for well-known CRC-associated species *F. nucleatum* (Piccinno et al., 2025).

To validate the generalisability of these results, the genus level abundance and prevalence of the ES CRC-associated taxa were evaluated *via* analyzing 16S rRNA gene amplicon sequencing from independent validation cohorts from Japanese populations. As expected, the prevalence of the CRC-associated genus was highly fluctuating among groups and cohorts except genus *Fusobacterium* (Fig. S5B). This emphasises the importance of expanding the cohort size to better explore the factors determining prevalence. The genus *Lancefieldella* was slightly enriched in ES CRC for small proportion of Cohort2, and genus *Schaalia* was enriched in precancerous stage of Cohort 3 (Fig. S5C). In contrast, *Solobacterium* was enriched in later-stage CRC for all cohorts, similar to *Fusobacterium* (Fig. S5C). These suggested the part of results could be generalized with independent cohorts from Japanese populations.

Although there are room to disentangle the heterogeneity in the presence and abundance ES CRC-associated species, several studies support the shotgun metagenomic insights of this study. A previous study suggested that So (formerly known as *Actinomyces odontolyticus*) can induce colonic dysplasia by releasing membrane vesicles and disrupting oxygen metabolism (Miyakawa et al., 2024). Sm was reported to selectively adhere to CRC cell lines *via* a CnaB-type domain-containing protein and to promote CRC progression (Chen et al., 2025). Notably, although it was not significant after multiple testing correction, So and Sm showed an enrichment trend in Stage 0 (*p* = 0.00760 for So, *p* = 0.0122 for Sm, respectively).

Given their relevance to CRC onset, we focused on Lp, So, and Sm as ES CRC-associated communities.

### Isolation and comparative genomics of ES CRC-associated species

Metagenomic analysis using MetaPhlAn4 provides insights into species-level genome bins (SGBs), and the results suggest that a specific Lp bin is involved in Stage 0 CRC (Fig. S7). Moreover, the SGB classification differed in Lp (Fig. S7A), and the type strain assigned to SGB964 was rarely observed in both normal and Stage 0, whereas the clinical strain assigned to SGB966 was prevalent (Fig. S7). These findings highlight the importance of strain-level investigations for understanding the effects of CRC-associated species. Therefore, we isolated clinical strains of Lp, So, and Sm (denoted by Lp_C, So_C, and Sm_C, respectively) from CRC patient faecal samples with antibiotic-supplemented selective medium and obtained complete genomes (Fig. S1B, Table S5). Together with the type strains Lp (JCM 10300, denoted by Lp_T), So (JCM 14871, So_T), and Sm (JCM 10645, denoted as Sm_T), the clinical strains were characterized. To validate the taxonomy of isolates, the clinical strains were confirmed to be classified as Lp, So, and Sm using 16S rRNA gene homology criteria (Kim et al., 2014) and unique clade-specific marker genes from the MetaPhlAn4 database (Fig. S7A). The genomic similarity measured by the average nucleotide identity (ANI) was inconsistent among species (Fig. S6B). The Lp and So genomes were diverse, with ANI values below the species threshold of 95 (Jain et al., 2018), suggesting that the clinical strains might have accumulated mutations and evolved. In contrast, Sm genomes showed high genomic homogeneity among strains, meeting the species requirement (ANI > 95), while the genome size of the clinical strain was 0.6 Mb smaller than that of the type strain, indicating a different course of acquiring genomic diversity from Lp or So.

**Figure 4:**
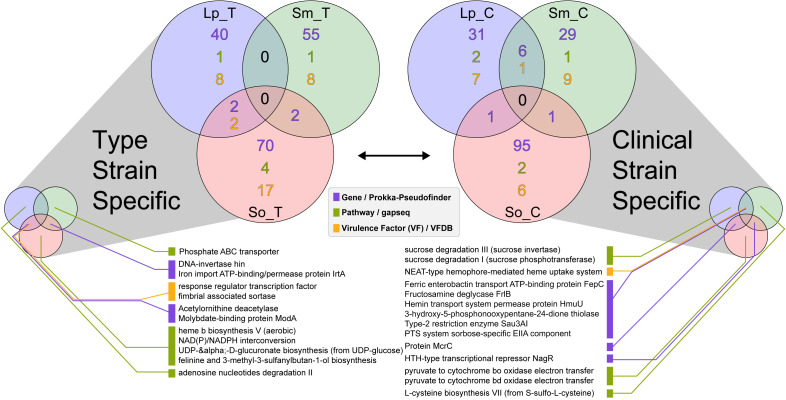
Strain specific genes, virulence factors, and pathways between type and clinical strains. The Venn diagram shows the observed numbers of each component. The right and left represent genes uniquely identified in the type and clinical strains, respectively. Purple: genes annotated by Prokka and screened by Pseudofinder; green: pathway annotated by gapseq; yellow: virulence factors annotated by VFDB with DIAMOND.

Subsequently, we conducted comparative genomics of their genes or pathways and characterized their functions only in type or clinical strain genomes (Fig. 4, Fig. S8). In the type and clinical strains, iron-related virulence factors were detected, indicating divergent iron acquisition or metabolism among the strains (Fig. S8). Iron-related functions, such as the haemophore-mediated haem uptake system, the ferric enterobactin transport ATP-binding protein, and the hemin transport system permease protein, were shared among clinical strains (Fig. 4). Anaemia and bleeding-related symptoms are common at CRC onset (Edna et al., 2012; Hreinsson, Jonasson & Bjornsson, 2014) and might supply iron to ES CRC-associated species. Therefore, iron-related genes might be important for the clinical strain adaptation of ES CRC-associated species. A previous study reported that So has a CnaB-type domain-containing protein that adheres to cancer cells, and this binding promotes CRC (Chen et al., 2025). We assessed the Cna and its domain by Prokka, DIAMOND with VFDB (Liu et al., 2022), and HMMER with Pfam. CnaB-type domain-containing protein-related components were detected in Lp and Sm across the two databases (Fig. S9A). To assess the enrichment of this domain in ES CRC-associated species, we collected complete and reference genomes from NCBI Refseq, and the CnaB-type domain was enriched in ES CRC-associated species (Fisher’s exact test, *p* = 0.031; Fig. S9B). This implies that ES CRC-associated species use a shared mechanism to interact with epithelial cells *via* a common adhesin. In summary, we isolated and collected types and clinical strains of Lp, Sm, and So, which harbour genetic heterogeneity and may be advantageous for nutrient acquisition, whereas they share a specific domain that adheres to CRC tissue.

### ES CRC-associated clinical isolates promote tumorigenesis

To address the effects of clinical strains on CRC tumorigenesis, different ES CRC-associated strains were orally administered to C57BL/6J ApcMin^/+^ mice (Fig. S1C). In this study, we prepared two distinct cocktails, namely, the type strains Lp_T, So_T, and Sm_T (denoted by Type_3Mix) and the clinical strains Lp_C, So_C, and Sm_C (denoted by Clinical_3Mix). The medium and *F. nucleatum* (Fn) were used as negative controls and positive controls, respectively. Ampicillin, neomycin, and vancomycin were added to the drinking water prior to bacterial treatment. The strains were directly ingested into the gut with an oral sonde 3 times/week for 5 weeks. Initially, seven, seven, eight, and seven mice (*N* = 29) were prepared for the control, Type_3Mix, Clinical_3Mix, and Fn groups, respectively, without weight differences (Kruskal–Wallis test, *p* > 0.05; Fig. S10).

**Figure 5:**
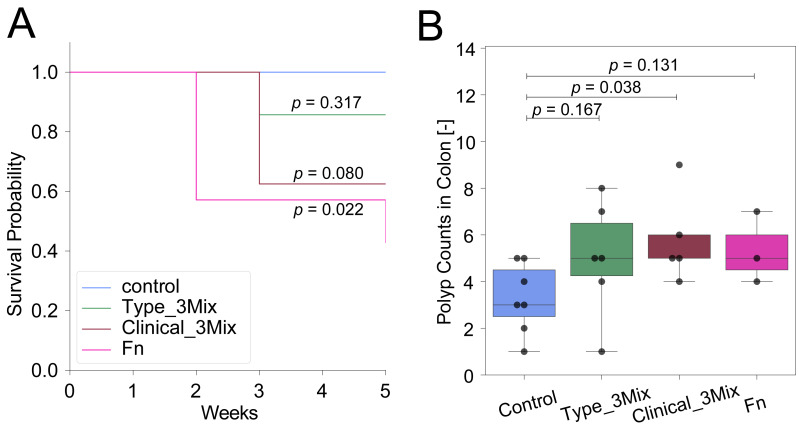
Strain-specific effects on mouse survival and polyp counts in the colon. (A) Kaplan–Meier curves, tested by the log-rank test. (B) Effects of strain inoculation on polyp counts in the colon, as determined by the Mann–Whitney U test.

To assess the effects of the isolates administration on mouse survival, survival was evaluated with Kaplan–Meier curves (Fig. 5A). As expected, the survival time of the Fn group was significantly shorter than that of the control group (log-rank test, *p* = 0.022). Similarly, the survival time in the Clinical_3Mix group was shorter than that in the control group, but the difference was not significant (*p* = 0.080). The effect of the Type_3Mix challenge on survival time was smaller than that of the Fn and Clinical_3Mix challenges.

To assess the tumorigenic effects of each group, we fixed the intestines of the mice in 10% neutral formalin buffer solution and counted the polyps in the colons after administration (Fig. 5B, Fig. S11). Owing to the genetic predisposition of the mice used, we observed polyps in the control groups. In the Isolated_3Mix group, the observed polyp count increased significantly (Mann–Whitney U test, *p* = 0.038). Similarly, the Type_3Mix and Fn groups showed increasing trends in polyp counts (not significant). These results highlighted differences in the strain-dependent effects. In contrast, no increasing trend was observed in the small intestine, suggesting a colon-specific effect of the group on tumorigenesis (Fig. S12). In summary, we observed clinical strain-specific effects on survival time and tumorigenesis, and compared with the type strains, the cocktail consisting of the ES CRC-associated clinical strains was potentially more pathogenic.

## Discussion

We aimed to infer the ES CRC-associated gut microbial community, understand the strain diversity, and assess its effect on tumorigenesis. We found that Lp was the most significant signature of ES CRC and correlated with other ES CRC-associated taxa, such as *Actinomyces* and *Solobacterium*. To understand the strain and functional diversity, we isolated strains of Lp, So, and Sm as ES CRC-associated community members and determined their complete genomes. Analysis of their average nucleotide identity revealed substantial heterogeneity (ANI < 90) between the types and clinical strains of Lp and So, despite being classified as the same species using 16S rRNA gene and clade-specific marker gene approaches. Comparative genomics suggests that the functional diversity within a species may differ in terms of nutrient acquisition and effects on CRC tumorigenesis. The introduced cocktail derived from clinical strains shortened the survival period of the CRC mouse model and significantly promoted tumorigenesis, suggesting a strain-specific mechanism in the gut of ES CRC patients.

Lp, Sm and So were suggested as CRC-associated species in early studies. Lp is known as an oral cavity-related bacteria (Copeland et al., 2009) and was originally reported to be associated with ES CRC (Yachida et al., 2019). This species was reported to mediate other H_2_S producers and to induce pancolitis, potentially *via* the H_2_S pathway (Mottawea et al., 2016). Previous studies have confirmed that an increased amount of H_2_S is exhaled from CRC patients (Du et al., 2024) and enriched in CRC tumor tissue, altering the immune response (Yue et al., 2023). Additionally, many gut commensals are involved in sulfur metabolism with dissimilatory sulfite reductases (dsr) or anaerobic sulfite reductase (asr) (Wolf et al., 2022). These genes were not identified in the So or Sm genomes, suggesting the presence of other possible interactions with Lp. So has been reported as an oral disease and CRC-associated species (Barrak et al., 2020; Wirbel et al., 2019; Yachida et al., 2019; Yu et al., 2017) and was recently shown to potentially promote CRC progression *via* the Integrin α2/β1-PI3K-AKT-mTOR-C-myc signaling pathway through selective adhesion to cancer cells (Chen et al., 2025). This mechanism could involve the CnaB-type domain as an adhesin to cancer cells, and this domain was identified in Lp and Sm (Fig. S8), suggesting the possibility of a shared mechanism promoting CRC progression across the ES CRC-associated species. So has also been described as a CRC-associated species (Konishi et al., 2022; Yachida et al., 2019). This species was previously described as a CRC-initiating species with secreted membrane vehicles and disruptive effects on mitochondria (Miyakawa et al., 2024).

Although these experimental validations of CRC have been conducted, the aforementioned studies of Lp, So, and Sm used only type strains, whereas strain diversity could play a crucial role in disease development. A cross-sectional study of IBD patients isolated clinical strains (Hall et al., 2017). The strain-resolved phylogeny and functions revealed that *Mediterraneibacter gnavus* shows phylogenetic dependence on disease states, and the IBD-related clade may harbour genes involved in oxidative stress responses, adhesion, iron acquisition, and mucus utilization in response to IBD-related changes in the gut environment (Hall et al., 2017). In this study, consistent with this report, the shared iron-related functions were characterized through comparative genomics. Anaemia and bleeding-related symptoms are common symptoms of CRC onset (Edna et al., 2012; Hreinsson, Jonasson & Bjornsson, 2014), and the resulting increase in iron influx by bleeding might facilitate the colonization of ES CRC-associated species. Indeed, the role of iron in bacterial pathogenesis has been well established (Neilands, 1995). Another cross-sectional study of CRC patients isolated clinical strains of *Bacteroides fragilis* and identified toxin gene *bft2*, a putative CRC marker, was enriched in isolates from CRC patients compared to those from controls (Rezaei et al., 2025). These studies underscore the importance of clinical specimens and the isolation process to explore the pangenome beyond type strains.

Moreover, most previous studies focused on the host–bacterium relationship and tended to consider monophyly. A machine learning-based study on CRC populations successfully described them into four distinct subgroups, and each subset had a unique microbial distribution (Rynazal et al., 2023). The community effects of gut bacteria have been considered in therapeutic fields, and a previous study demonstrated the efficacy of designed consortia in a colitis setting (Van Der Lelie et al., 2021). Likewise, we examined the effects of ES CRC-associated species cocktails on a CRC mouse model and observed clinical strain-specific pathogenesis on survival and tumorigenesis.

The present study has several limitations, including (1) the limited accessibility to validation cohorts, (2) the limited number of examined species, isolated strains, and their combinations present in the community, (3) the lack of mechanistic perspectives, and (4) the limitation arising from the use of a model that spontaneously develops adenoma. To validate the ES CRC markers identified in this study, independent cohorts are required. Given the geographical dependency of gut microbiome (Andreu-Sánchez et al., 2025), and the uniqueness of the Japanese population (Nishijima et al., 2016), these validation cohorts should be recruited in Japan and include patients with ES CRC and precancerous lesions. However, such cohorts are unavailable for shotgun metagenomics and only available as 16S rRNA amplicon sequencing datasets analyzed in this study (Fig. S5). We identified other species that were positively correlated with Lp, such as *Mogibacterium diversum* and *Veillonella atypica*, that were enriched in ES CRC. Owing to the limited number of strains, there might be unexplored diversity among genomes and functions. Furthermore, synergistic effects could be driven by the combination of the strains as a community on CRC progression. In this study, the mechanisms underlying promoted tumorigenesis were unexplored in terms of the cross-feeding among bacteria or crosstalk with host tissue; thus, an *in vitro* co-culture experiment could help to understand the holistic bacteria-host interaction. Indeed, previous study demonstrated the cocultured CRC-associated species *F. nucleatum* and *Parvimonas micra* form biofilms synergistically (Horiuchi et al., 2020), and biofilms formed on CRC tissue have been observed in right-colon CRC (Dejea et al., 2014), which may contribute to increased pathogenesis. Ultimately, the effects of multiple combinations of strains on CRC should be generalized. This study employed the ApcMin^/+^ model under SPF conditions. Although this model is widely used to assess tumorigenesis and captures the early event of sporadic CRC, its metabolism differs from that in humans (Mestas & Hughes, 2004). In addition to survival and polyp counts, intratumor bacterial loads, immune profiles, and gene expression in host tissue should be addressed to elucidate the detailed mechanism of tumorigenesis.

## Conclusions

We inferred the ES CRC-associated community and strain-specific effects on tumorigenesis. The clinical strains, including Lp, Sm, and So, found in the gut of CRC patients were markedly different from the type strains and harboured distinct genes, virulence factors, and pathways, such as iron-related functions. Compared with the ApcMin^/+^ mice that received the type strains, the ApcMin^/+^ mice that received the clinical strains exhibited shorter survival times and greater polyp formation. These findings underscore the importance of considering the strain-dependent effects of gut commensals on CRC development, and further strains and their combinations need to be explored to understand the mechanisms driven by the gut microbial community.

## Supplemental Information

10.7717/peerj.21488/supp-1 Supplemental Information 1 Overview of this study (A) Detection of the early stage CRC-associated species by abundance comparison, and community by correlation analysis. (B) Isolation and comparative genomics of type and clinical strains. (C) Mice experiments for evaluationg the early stage CRC-associated effects on the polyp development.

10.7717/peerj.21488/supp-2 Supplemental Information 2 Enriched and depleted species in precancerous stage (multiple polyps) relative to normal Red: significantly associated species (FDR ¡ 0.1); grey: not significant.

10.7717/peerj.21488/supp-3 Supplemental Information 3 Inferred microbial community in Normal and Stage 0 The edge on the network shows the significant positive correlation among 2 species (p ¡ 0.01, correlation value by Fastspar ¿0.5). The red nodes: early stage CRC-associated species, blue: adjacent species to the early stage CRC-associated species, white: other species.

10.7717/peerj.21488/supp-4 Supplemental Information 4 The comparison of the number of positively correlated species (degree), tested by Mann-Whitney U test

10.7717/peerj.21488/supp-5 Supplemental Information 5 The prevalence and abundance of the ES CRC-associated species in Japanese cohorts (A) Prevalence of the ES-CRC associated species. (B) Prevalence, and (C) abundance of the ES-CRC associated genus in independent validation cohorts from Japanese populations. The abundance was estimated using publicly available 16S rRNA gene amplicon sequencing dataset. The statisical test for abundance was performed via Mann-Whitney U test. The genus Fusobacterium and the species Fusobacterium nucleatum were added as well-known representative of CRC-associated species.

10.7717/peerj.21488/supp-6 Supplemental Information 6 Relative abundance of Lancefieldella parvula-related SGB estimated by MetaPhlAn4

10.7717/peerj.21488/supp-7 Supplemental Information 7 The identification of the species with two independent annotation, and their heterogeneity (A) The species identification by 16S rRNA genes and unique clade-specific marker genes provided by MetaPhlAn4. (B) Average nucleotide identity among type and clinical strains.

10.7717/peerj.21488/supp-8 Supplemental Information 8 Differentially observed genes, virulence factors, and pathways for each species

10.7717/peerj.21488/supp-9 Supplemental Information 9 The identification of the CnaB-type domain (A) Identified genes with 3 distinct methods, Prokka with UniProtKB, DIAMOND with VFDB, and HMMER with Pfam. In the left panel, red cell: positive detection, blue: negative detection. In the right panel, the detailed distribution of the full length bit score estimated by HMMER search with Pfam. (B) The genome number harboring Cna B-type domain, and the domain enrichment in CRC-associated species.

10.7717/peerj.21488/supp-10 Supplemental Information 10 The mice weight during treatment periods and sex dependence, tested by Kruskal–Wallis test

10.7717/peerj.21488/supp-11 Supplemental Information 11 Fixed Tissues (A) Small intestines. (B) Colons. All tissues were pictured in the sterilized square petri dishes of the samp dimensions (140 mm × 100 mm)

10.7717/peerj.21488/supp-12 Supplemental Information 12 Effects of the community innoculation to mouse on the polyp counts in small intestine, tested by Mann-Whitney U test

10.7717/peerj.21488/supp-13 Supplemental Information 13 Supplemented antibiotics to isolate targeted species

10.7717/peerj.21488/supp-14 Supplemental Information 14 Comparisons of species relative abundance (MetaPhlAn4) between Normal and Stage_0 PV: prevalence, MD: median, MN: mean.

10.7717/peerj.21488/supp-15 Supplemental Information 15 The effects of confounders on the Normal and Stage 0 populations, analysed via MaAsLin3

10.7717/peerj.21488/supp-16 Supplemental Information 16 Comparisons of species relative abundance (MetaPhlAn4) between Normal and Multiple polyps (MP) PV: prevalence, MD: median, MN: mean.

10.7717/peerj.21488/supp-17 Supplemental Information 17 Genomic completeness, contamination, GC contents, genome size and coding density assessed by CheckM

10.7717/peerj.21488/supp-18 Supplemental Information 18 Lp_C_genome

10.7717/peerj.21488/supp-19 Supplemental Information 19 Sm_C_genome

10.7717/peerj.21488/supp-20 Supplemental Information 20 So_C_genome

10.7717/peerj.21488/supp-21 Supplemental Information 21 So_T_genome

10.7717/peerj.21488/supp-22 Supplemental Information 22 ARRIVE 2.0 Checklist

10.7717/peerj.21488/supp-23 Supplemental Information 23 Raw data - mouse weight

10.7717/peerj.21488/supp-24 Supplemental Information 24 Raw data - polyp size and count

## Additional Information and Declarations

### DNA Deposition

The following information was supplied regarding the deposition of DNA sequences: The raw sequence data are available at DDBJ Sequence Read Archive: SAMD01780340 (Lp_C), SAMD00902036 (So_T), SAMD00902037 (So_C), SAMD01780341 (Sm_C).

### Data Availability

The following information was supplied regarding data availability: The data is available in the Supplemental Files. The code is available at Zenodo: DSuzuki. (2025). yamada-lab/CRC_Associated_Community: 1.0.0 (1.0.0). Zenodo. https://doi.org/10.5281/zenodo.18013616. The raw sequence data are available at DDBJ Sequence Read Archive: SAMD01780340 (Lp_C), SAMD00902036 (So_T), SAMD00902037 (So_C), SAMD01780341 (Sm_C).
