# Supplementary material for: Clinical strains isolated from early-stage colorectal cancer patients promote tumorigenesis
Source: PeerJ. 2026 Jul 14;14:e21488. doi: 10.7717/peerj.21488 (PMC13378470; doi:10.7717/peerj.21488)
Supplement: Supplemental Information 1 — (A) Detection of the early stage CRC-associated species by abundance comparison, and community by correlation analysis. (B) Isolation and comparative genomics of type and clinical strains. (C) Mice experiments for evaluationg the early stage CRC-associated effects on the polyp development. [file peerj-14-21488-s001.pdf]

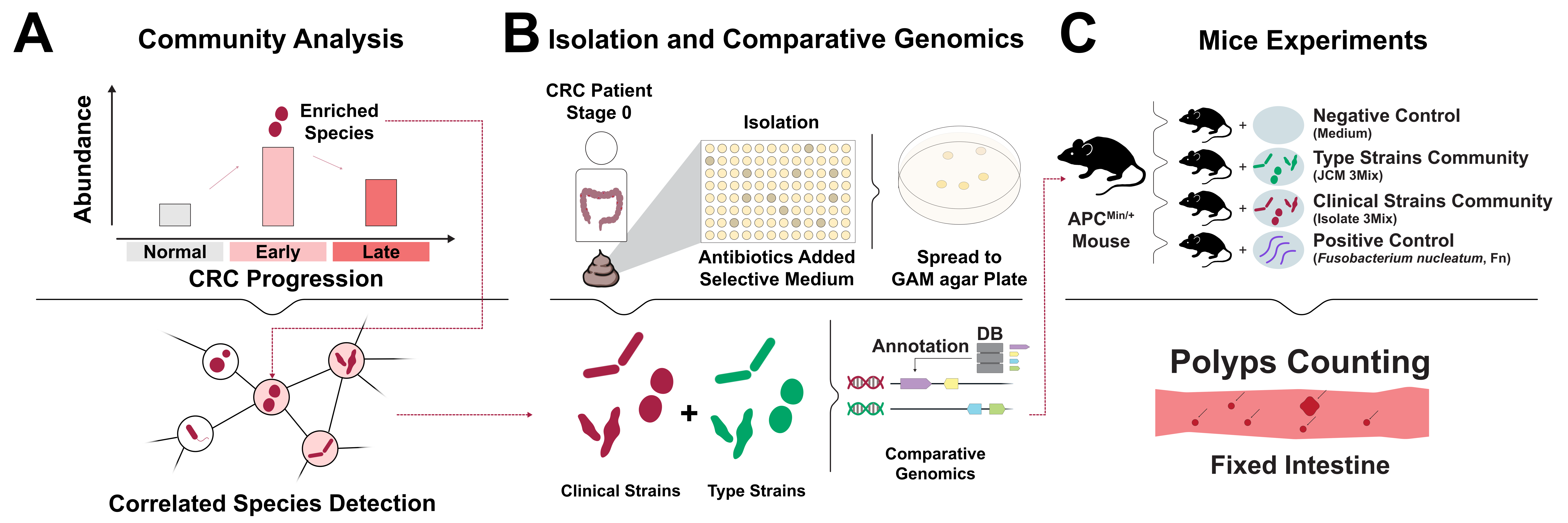
A
Community Analysis
Abundance
Enriched Species
Normal
Early
Late
CRC Progression
Correlated Species Detection
B
Isolation and Comparative Genomics
CRC Patient Stage 0
Isolation
Antibiotics Added Selective Medium
Spread to GAM agar Plate
Clinical Strains
Type Strains
Annotation
DB
Comparative Genomics
C
Mice Experiments
APCMin/+ Mouse
Negative Control
(Medium)
Type Strains Community
(JCM 3Mix)
Clinical Strains Community
(Isolate 3Mix)
Positive Control
(Fusobacterium nucleatum, Fn)
Polyps Counting
Fixed Intestine
