# Supplementary figures and images for: Clinical strains isolated from early-stage colorectal cancer patients promote tumorigenesis
Source: PeerJ. 2026 Jul 14;14:e21488. doi: 10.7717/peerj.21488 (PMC13378470; doi:10.7717/peerj.21488)

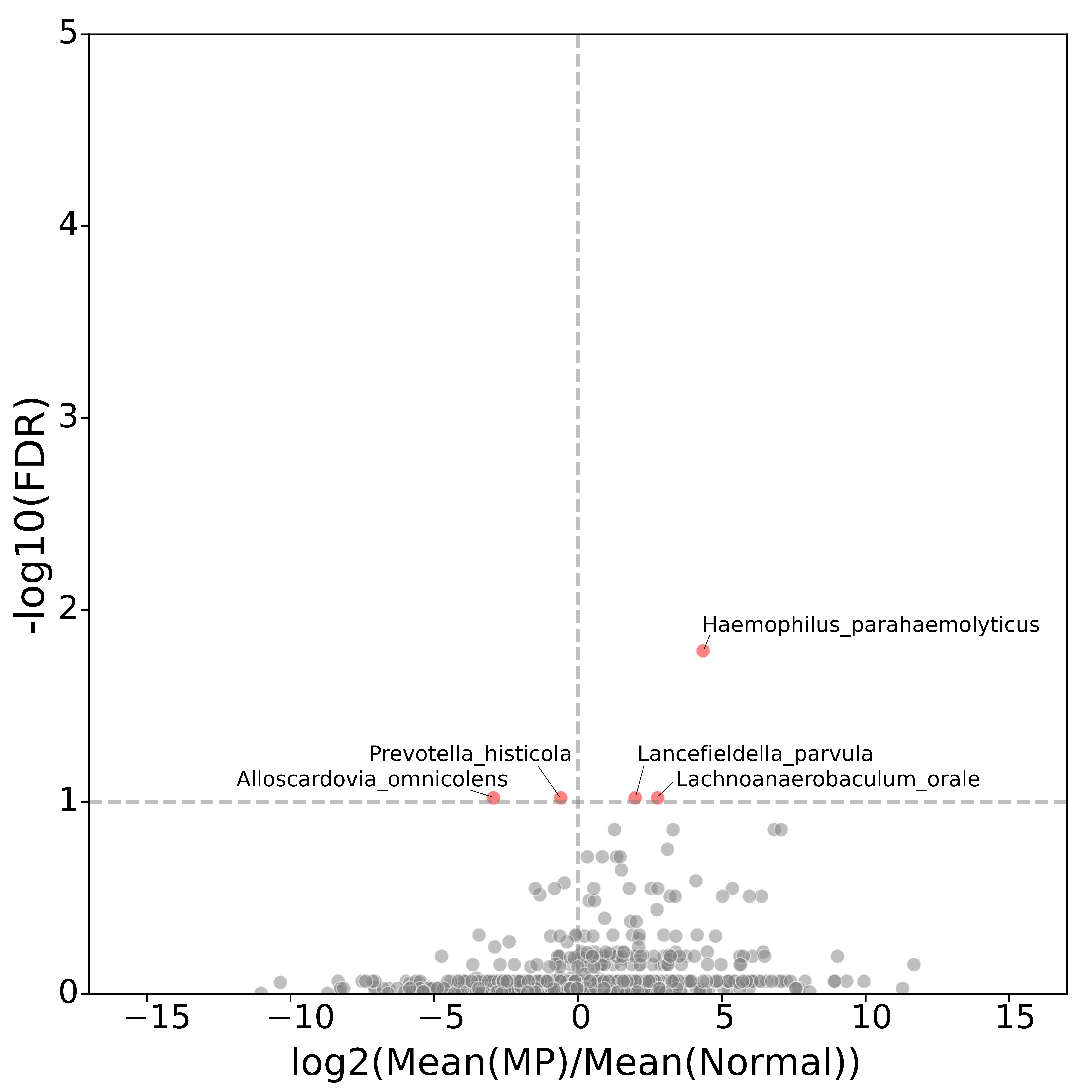

Supplement: Supplemental Information 2 — Red: significantly associated species (FDR ¡ 0.1); grey: not significant. [file peerj-14-21488-s002.pdf]

# Normal

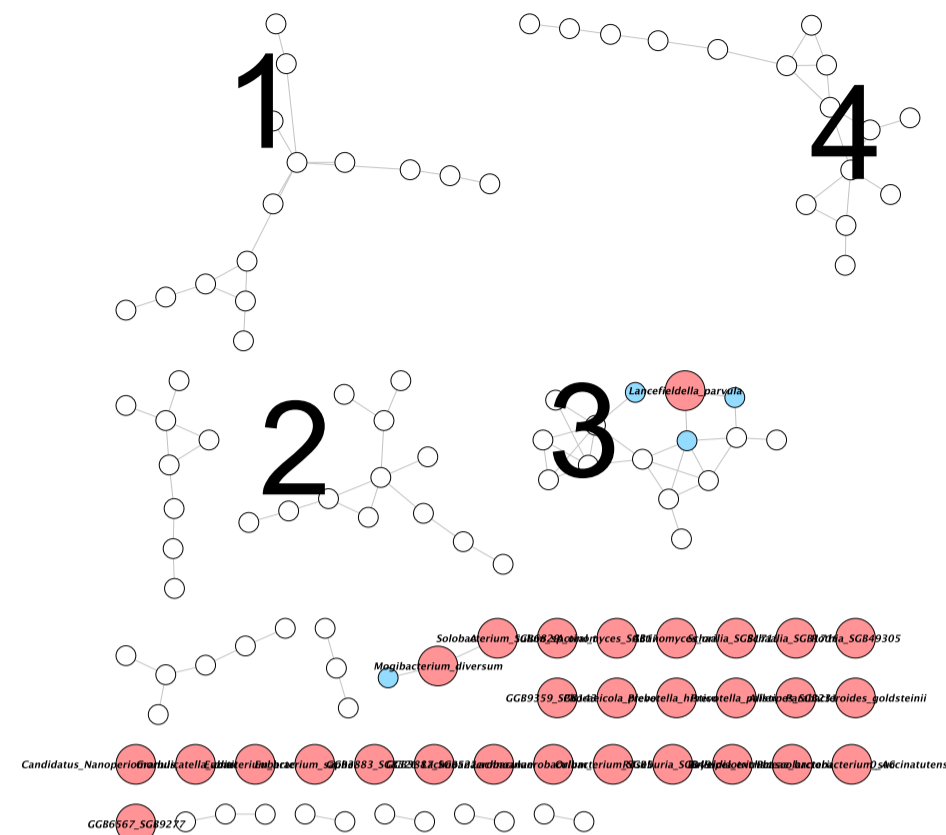

# Stage 0

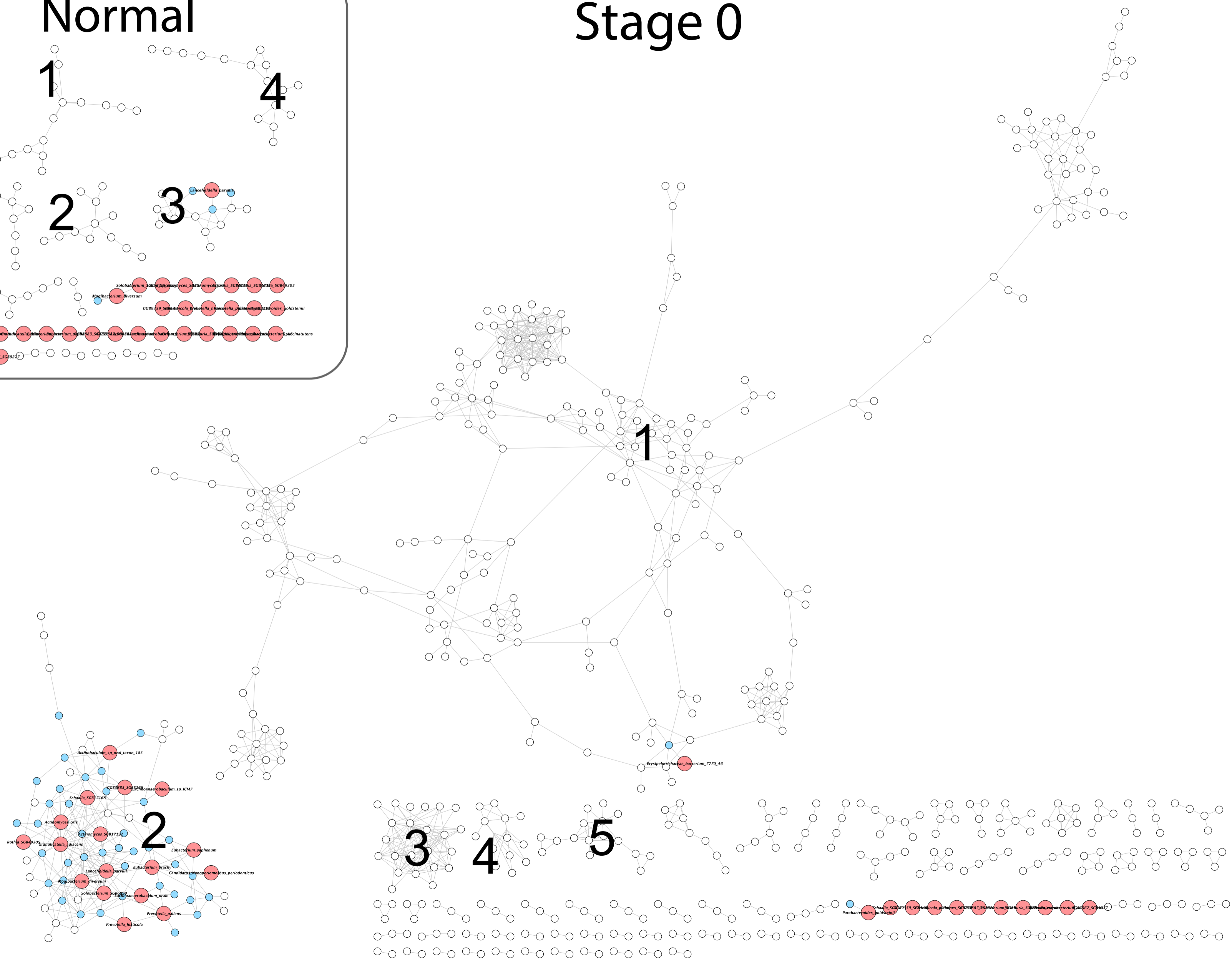

Supplement: Supplemental Information 3 — The edge on the network shows the significant positive correlation among 2 species (p ¡ 0.01, correlation value by Fastspar ¿0.5). The red nodes: early stage CRC-associated species, blue: adjacent species to the early stage CRC-associated species, white: other species. [file peerj-14-21488-s003.pdf]

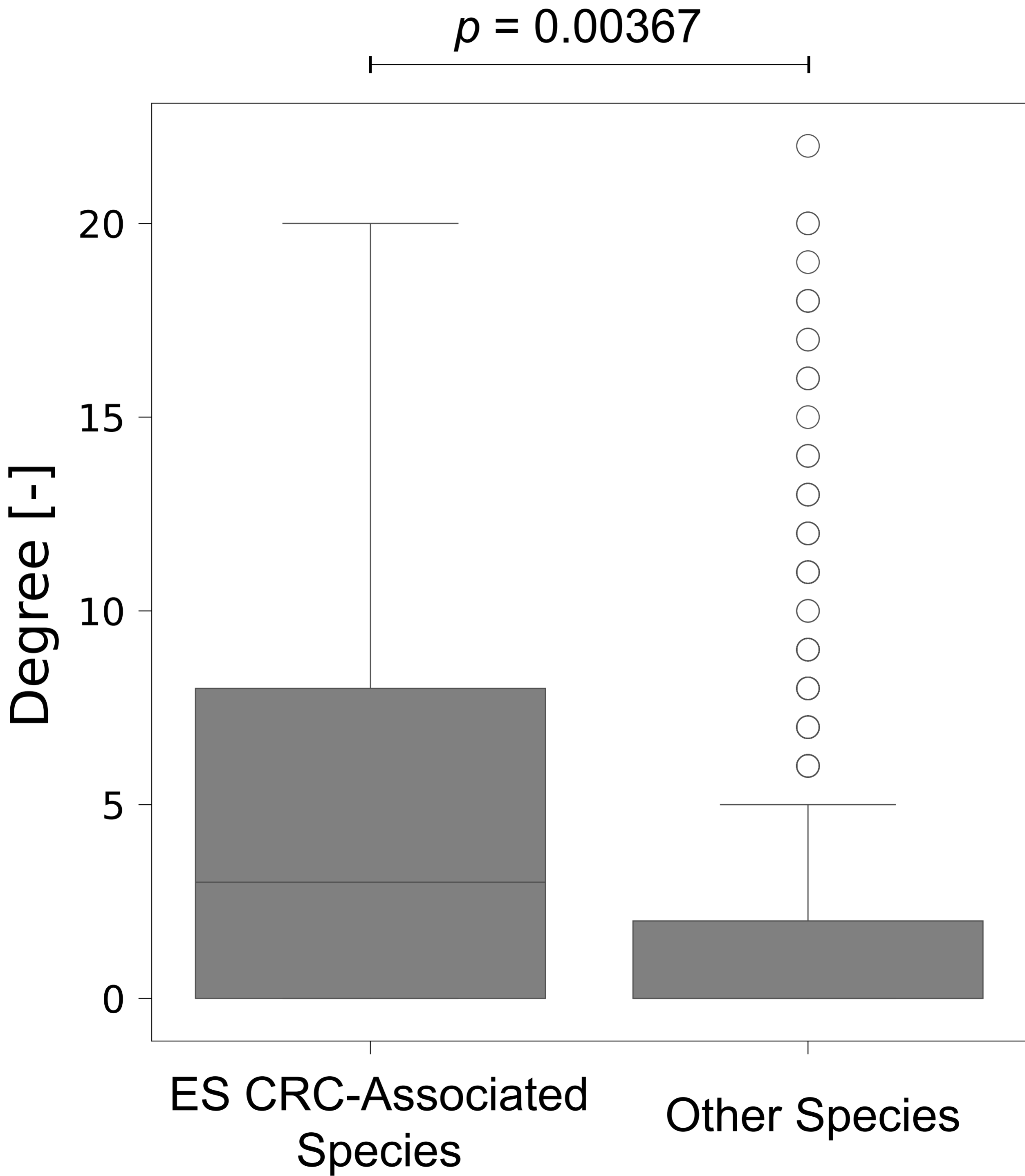

Supplement: Supplemental Information 4 [file peerj-14-21488-s004.pdf]

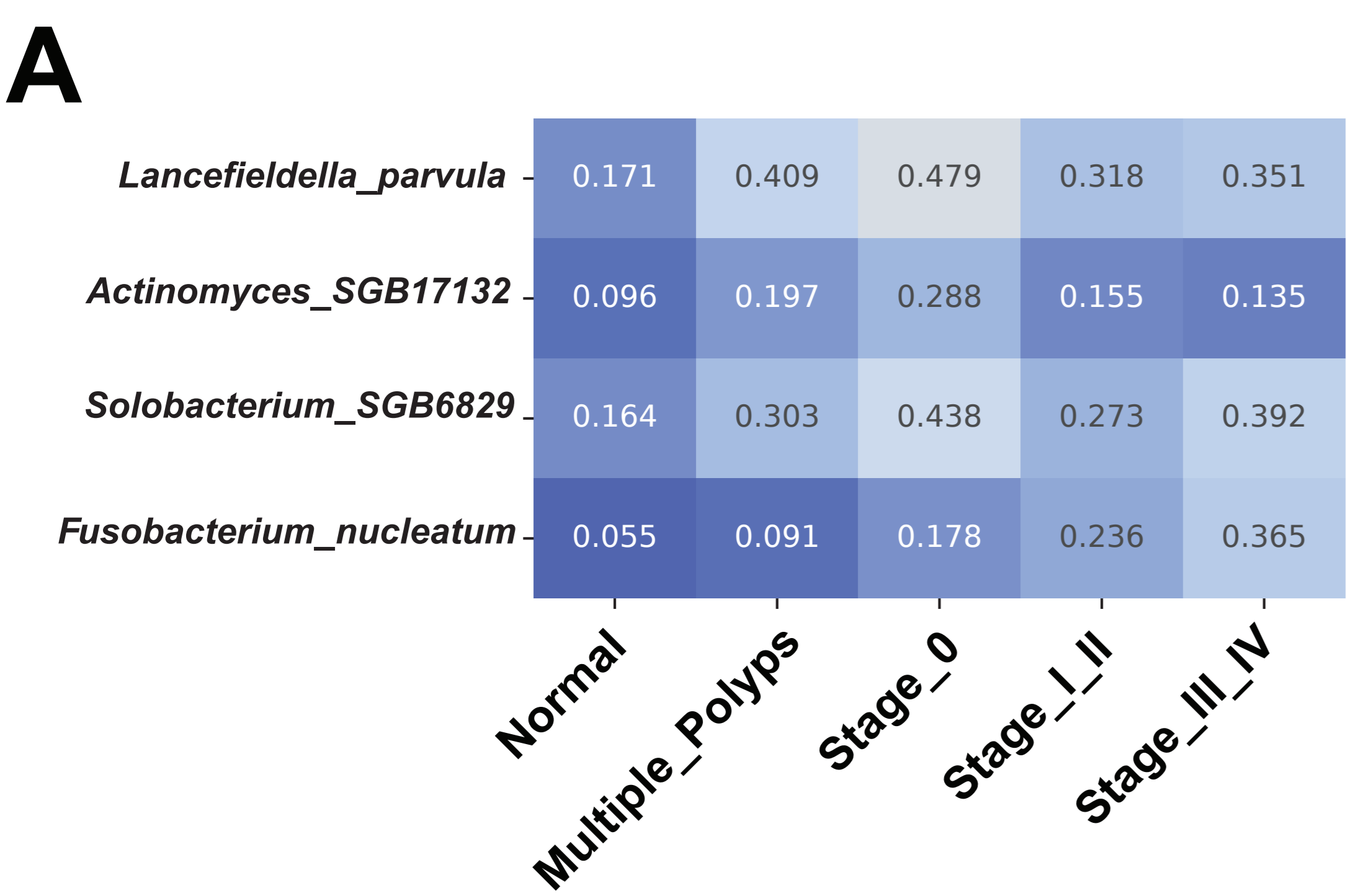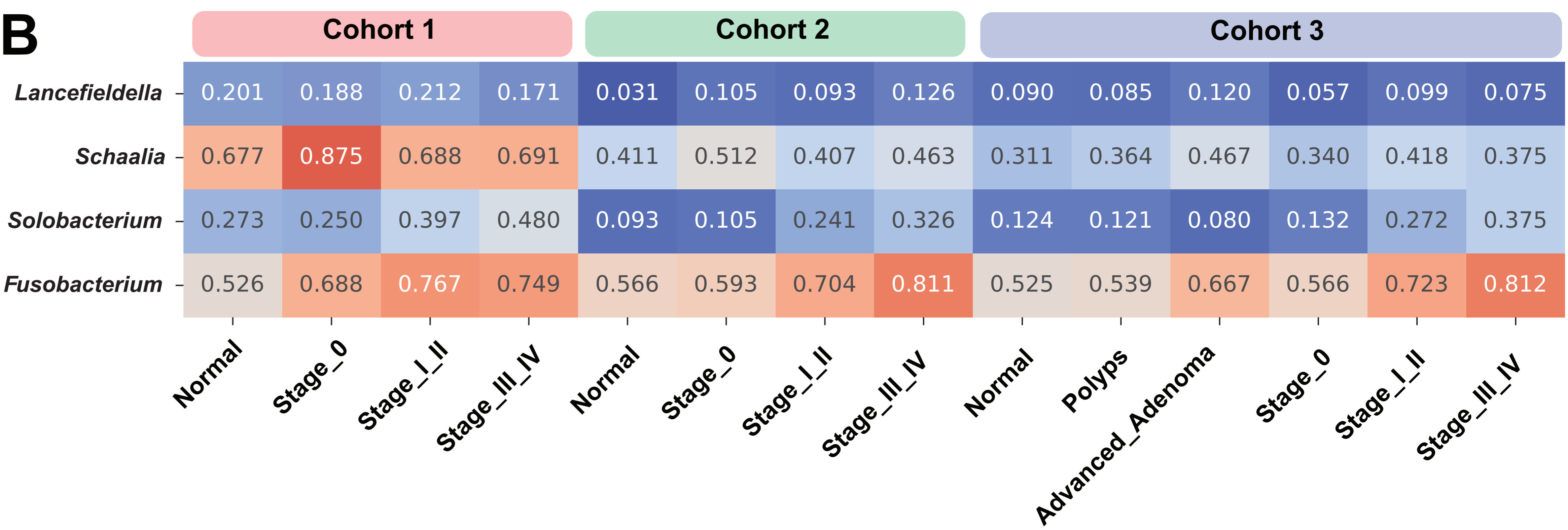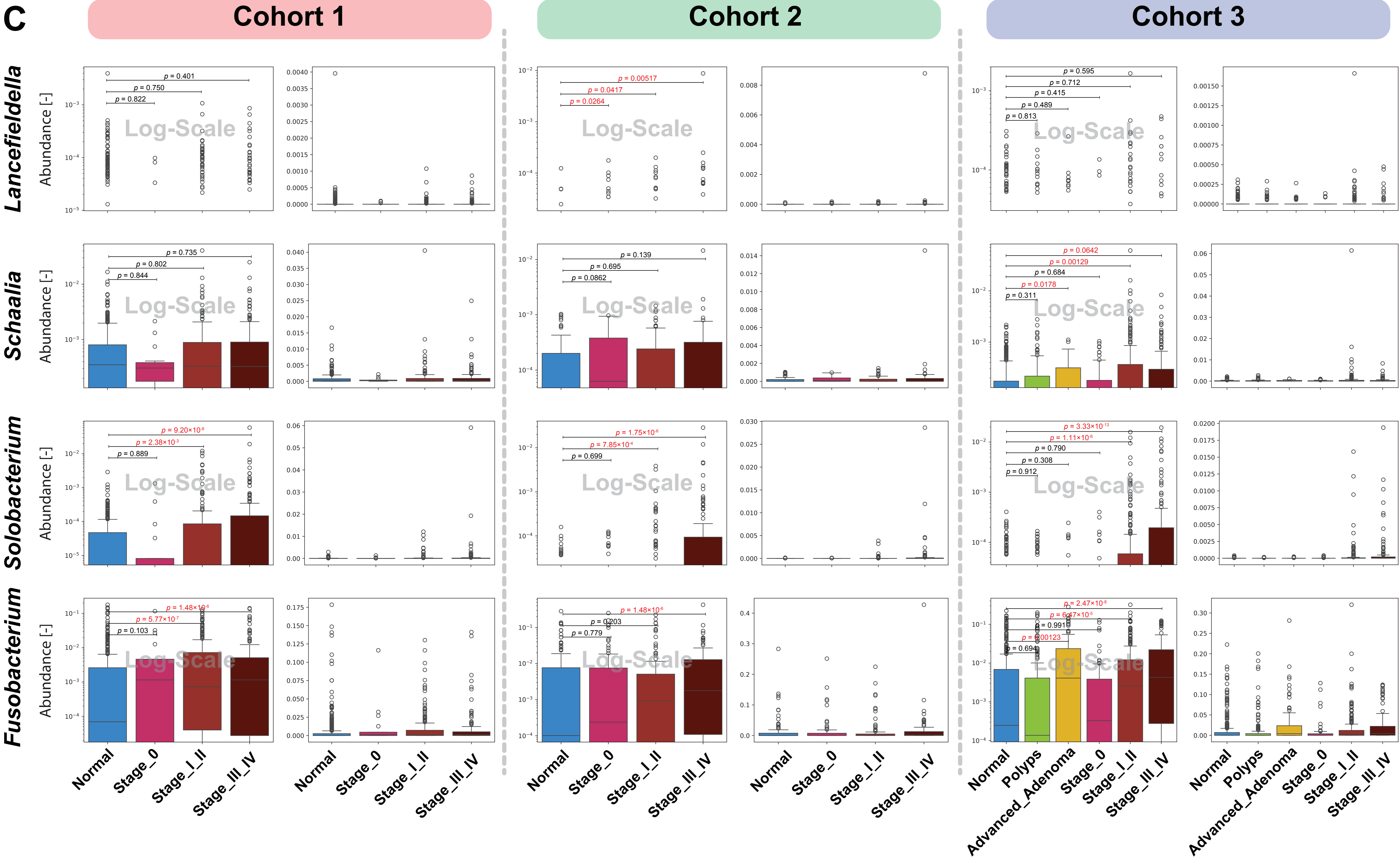

Supplement: Supplemental Information 5 — (A) Prevalence of the ES-CRC associated species. (B) Prevalence, and (C) abundance of the ES-CRC associated genus in independent validation cohorts from Japanese populations. The abundance was estimated using publicly available 16S rRNA gene amplicon sequencing dataset. The statisical test for abundance was performed via Mann-Whitney U test. The genus Fusobacterium and the species Fusobacterium nucleatum were added as well-known representative of CRC-associated species. [file peerj-14-21488-s005.pdf]

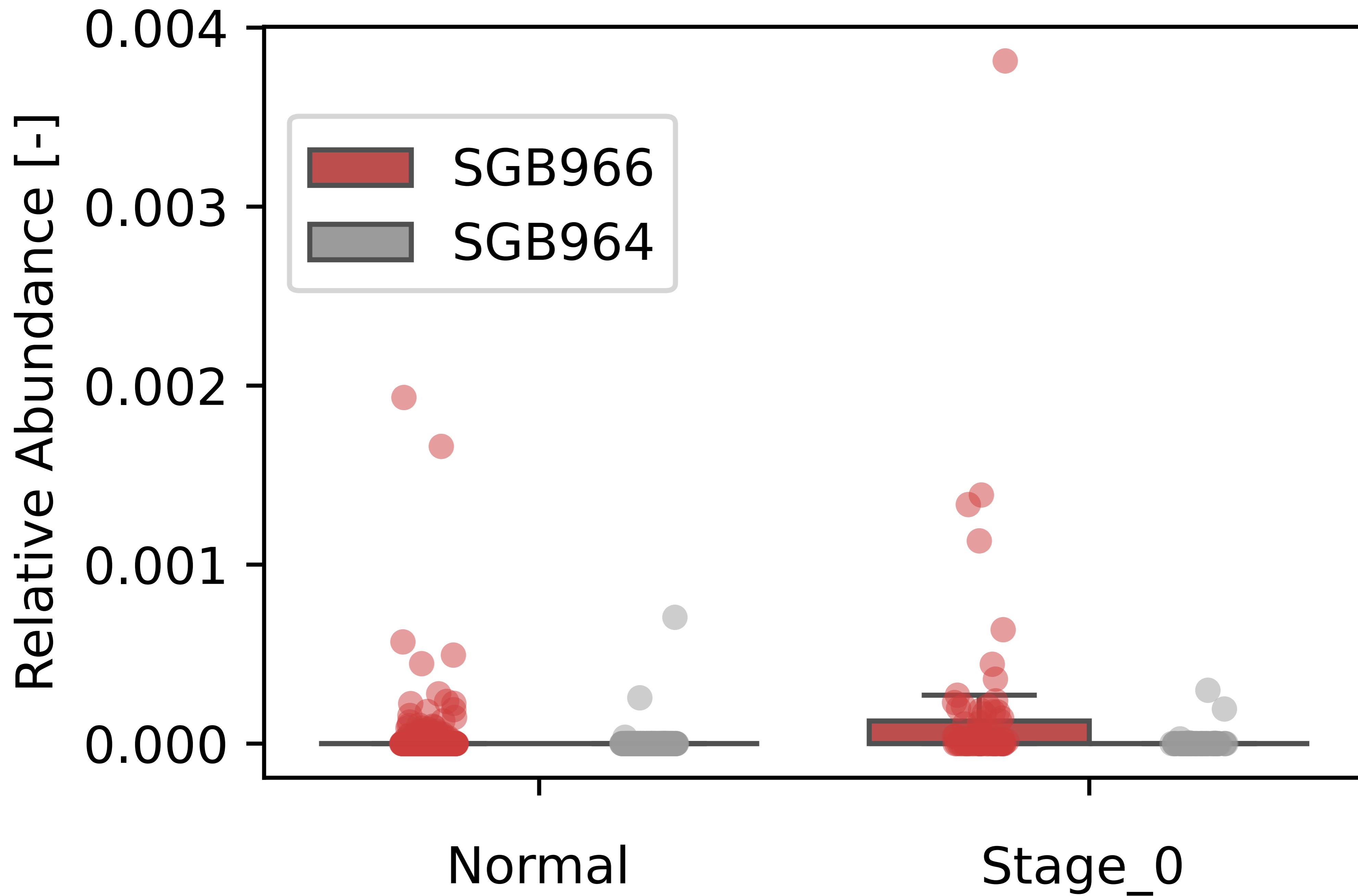

Supplement: Supplemental Information 6 [file peerj-14-21488-s006.pdf]

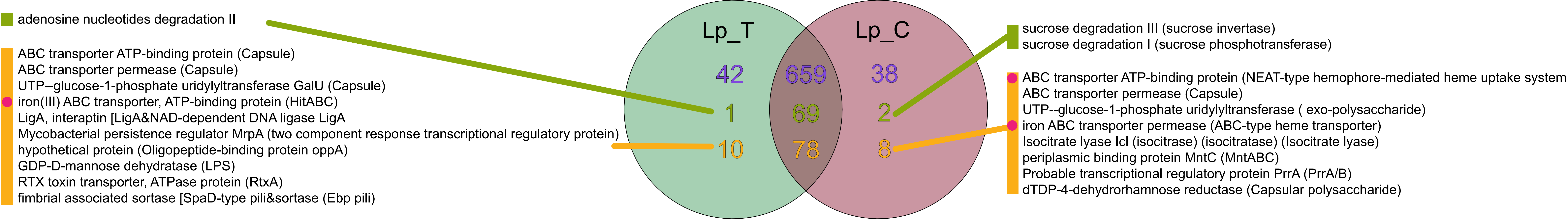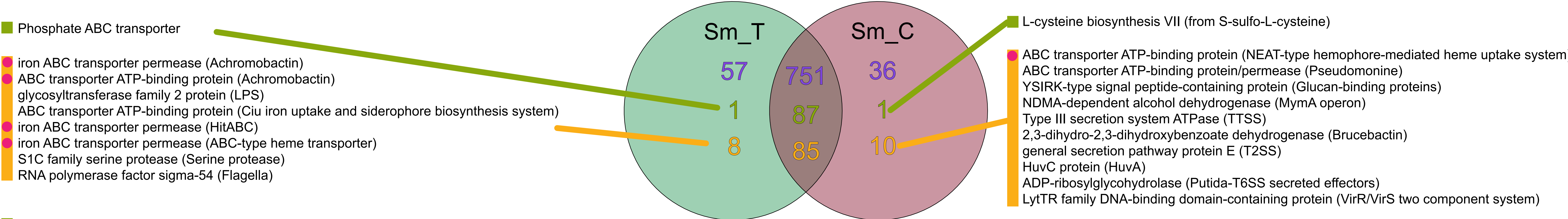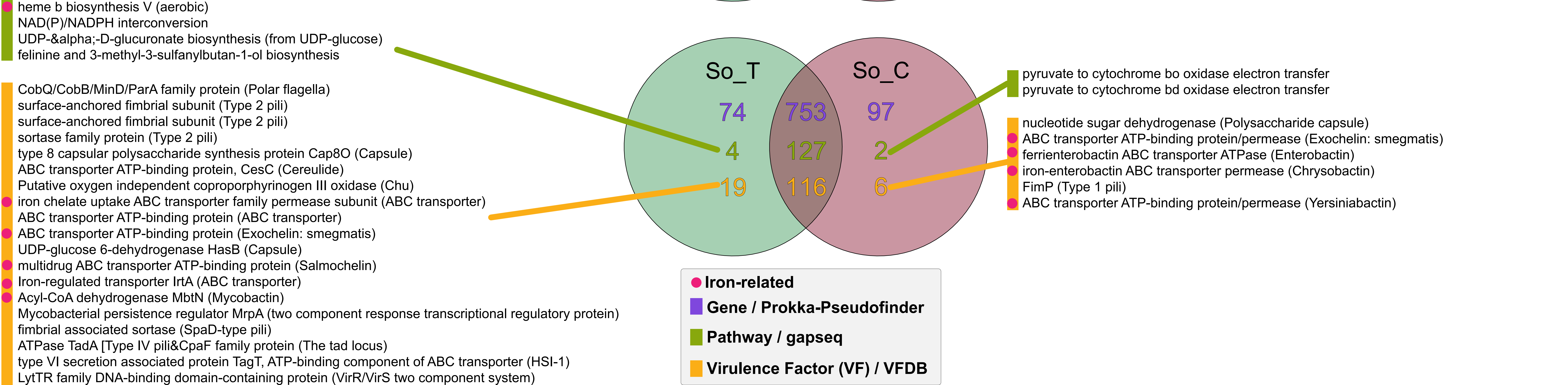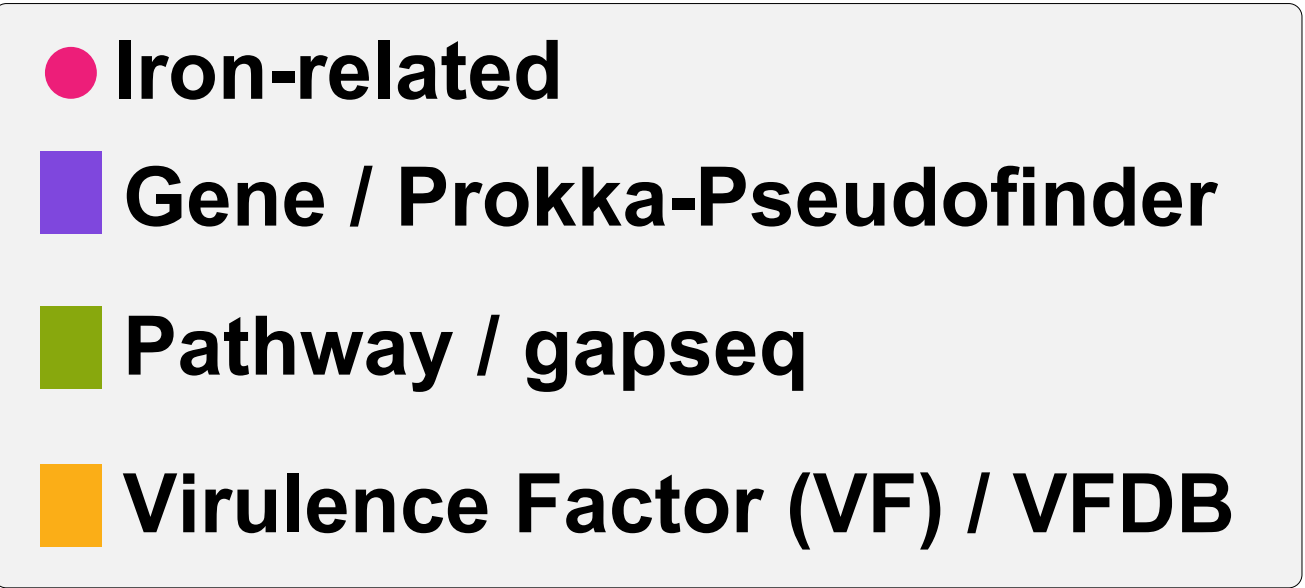

Supplement: Supplemental Information 8 [file peerj-14-21488-s008.pdf]

# A

Prokka/UniProtKB

DIAMOND/VFDB

HMMER/Pfam

Lp\_C

Lp\_T

Sm\_C

Sm\_T

So\_C

So\_T

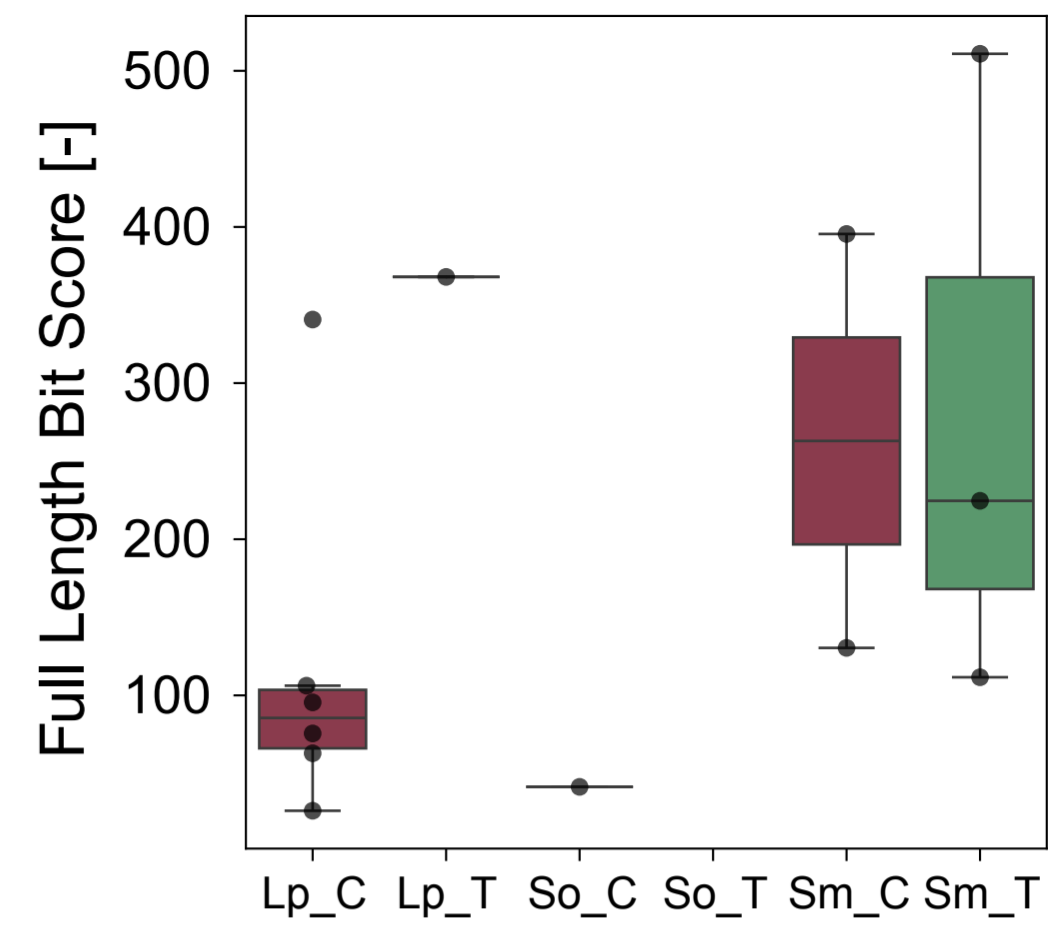

# B

$p_{\text{Fisher\_Exact}} = 0.03$

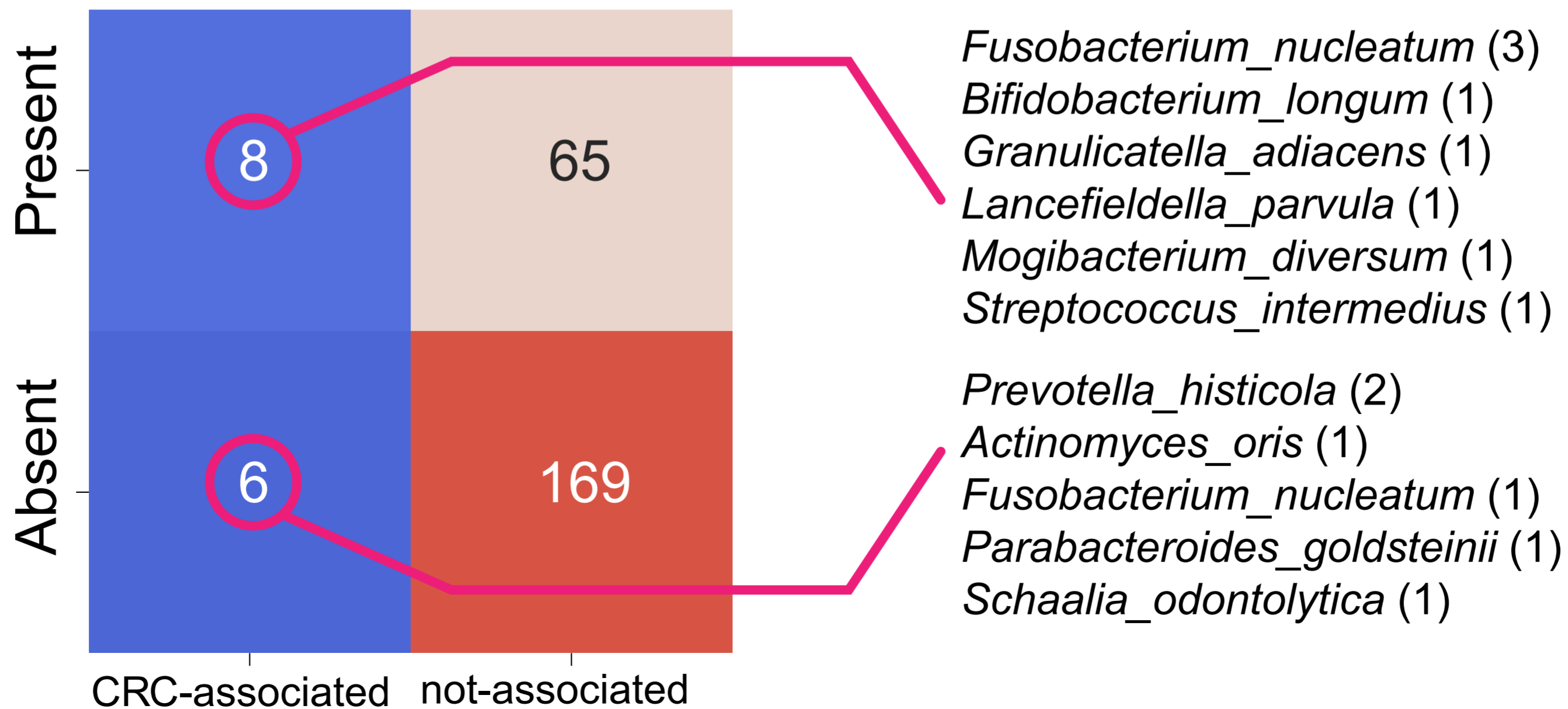

Supplement: Supplemental Information 9 — (A) Identified genes with 3 distinct methods, Prokka with UniProtKB, DIAMOND with VFDB, and HMMER with Pfam. In the left panel, red cell: positive detection, blue: negative detection. In the right panel, the detailed distribution of the full length bit score estimated by HMMER search with Pfam. (B) The genome number harboring Cna B-type domain, and the domain enrichment in CRC-associated species. [file peerj-14-21488-s009.pdf]

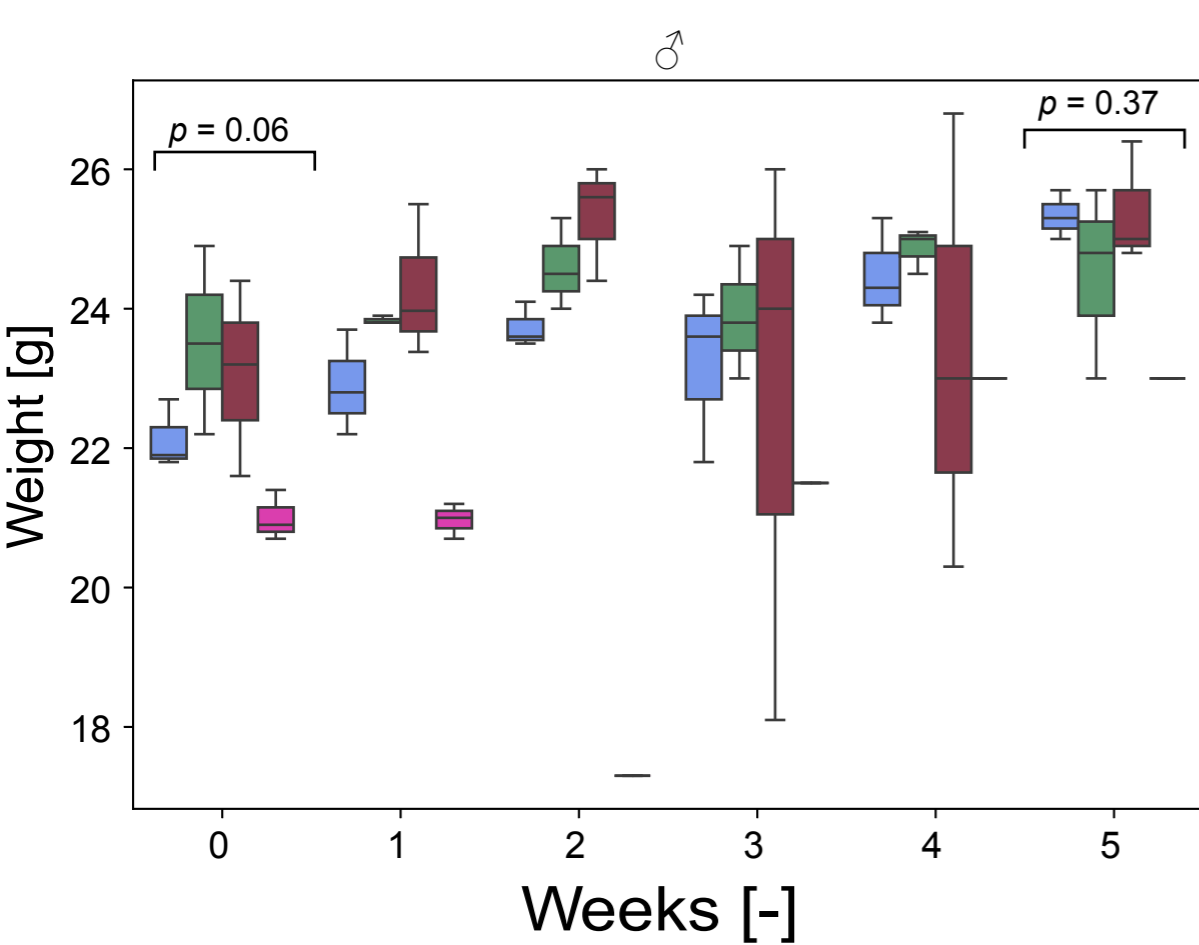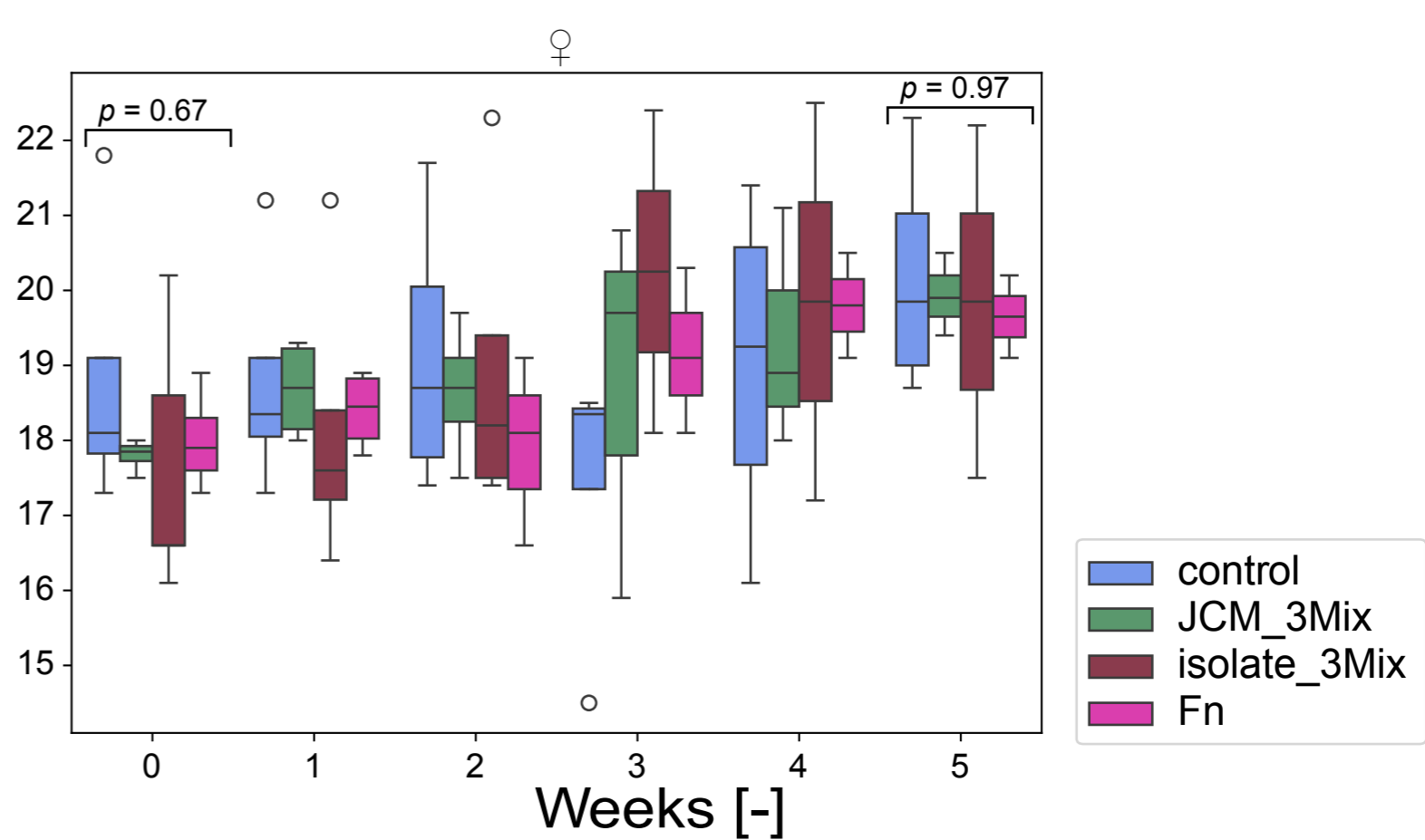

Supplement: Supplemental Information 10 [file peerj-14-21488-s010.pdf]

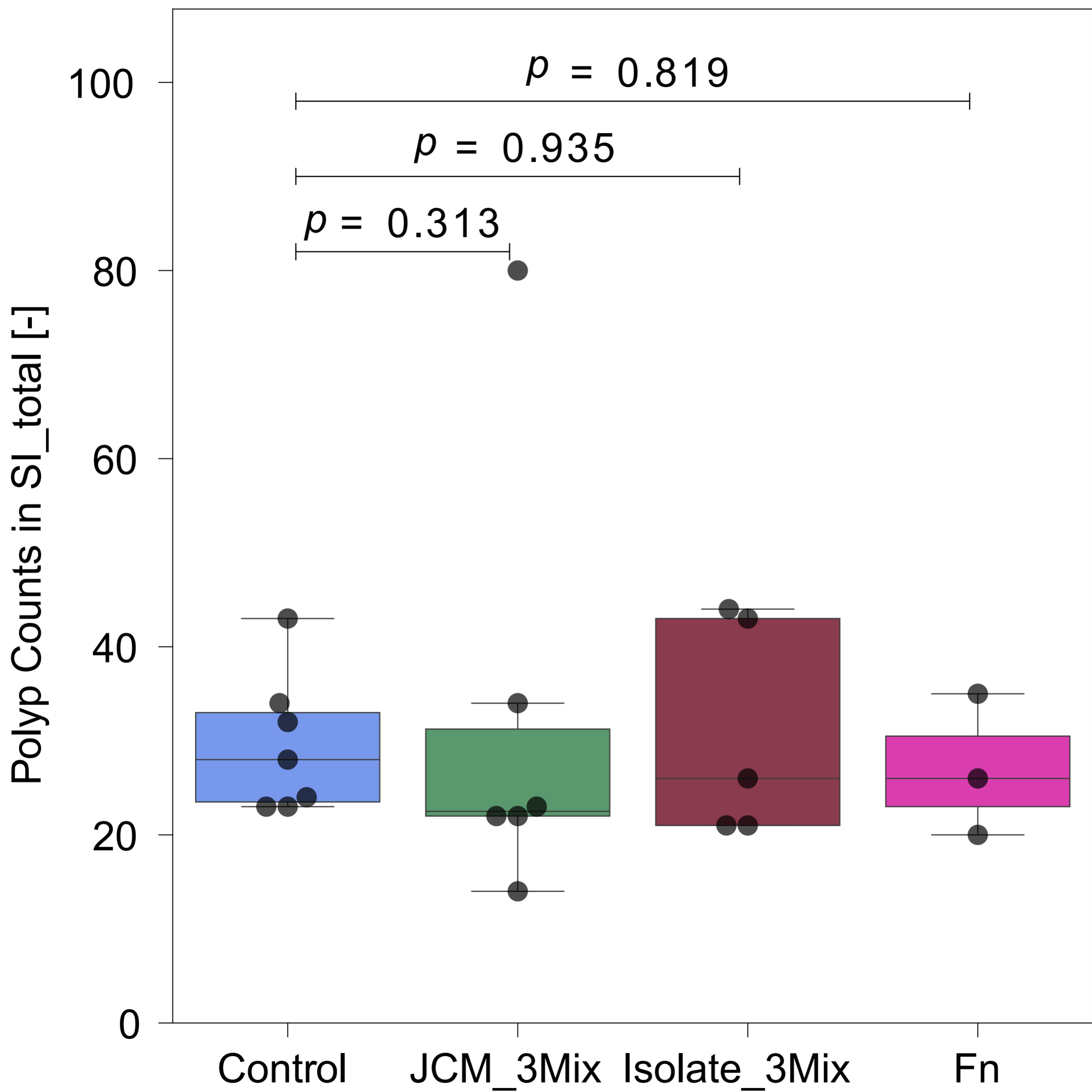

Supplement: Supplemental Information 12 [file peerj-14-21488-s012.pdf]
