# Supplementary material for: Clinical strains isolated from early-stage colorectal cancer patients promote tumorigenesis
Source: PeerJ. 2026 Jul 14;14:e21488. doi: 10.7717/peerj.21488 (PMC13378470; doi:10.7717/peerj.21488)
Supplement: Supplemental Information 7 — (A) The species identification by 16S rRNA genes and unique clade-specific marker genes provided by MetaPhlAn4. (B) Average nucleotide identity among type and clinical strains. [file peerj-14-21488-s007.pdf]

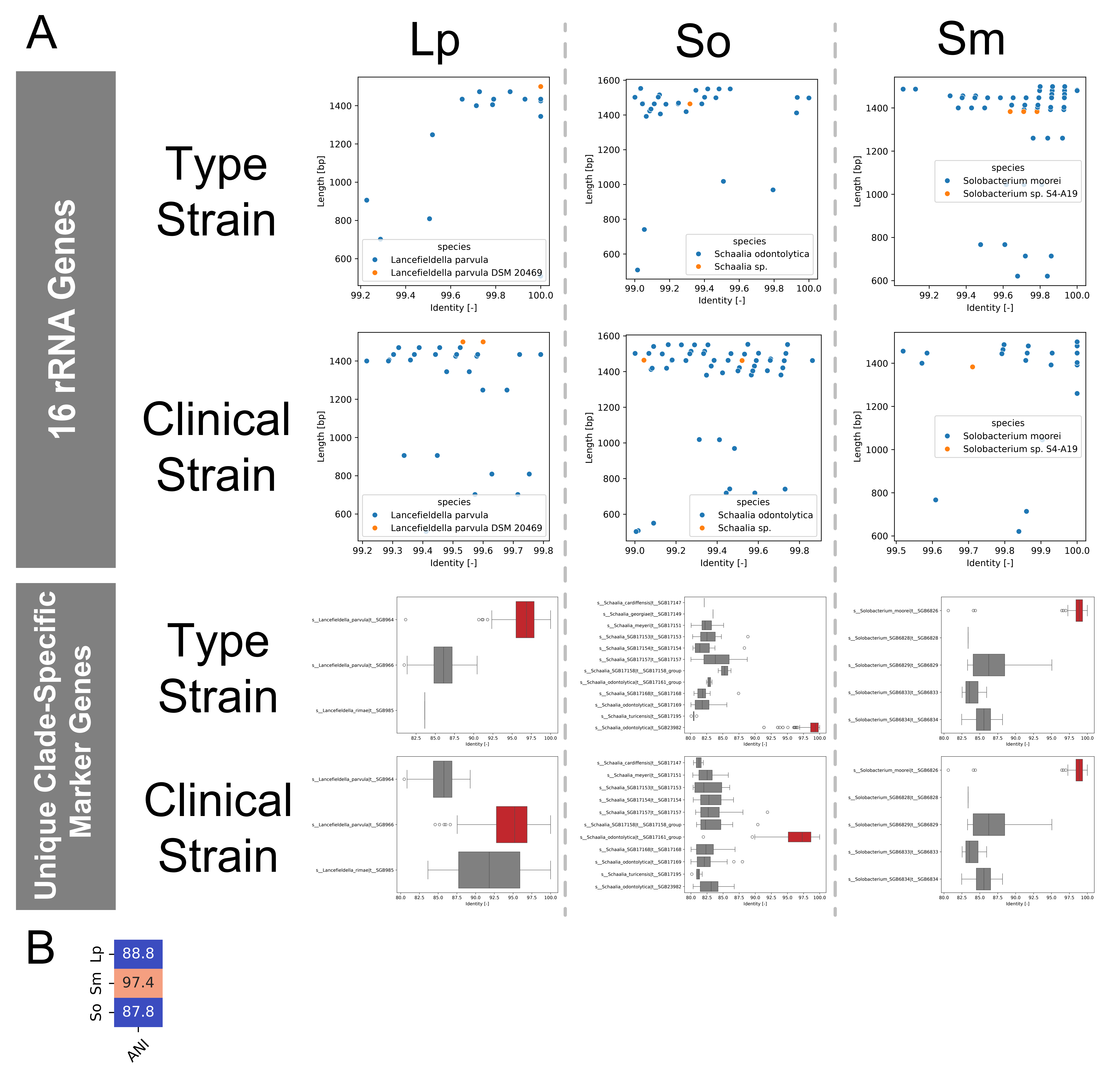

A
Lp
So
Sm
16 rRNA Genes
Type Strain
Clinical Strain
Unique Clade-Specific Marker Genes
Type Strain
Clinical Strain
Length [bp]
Identity [-]
species
Lancefieldella parvula
Lancefieldella parvula DSM 20469
Schaalia odontolytica
Schaalia sp.
Solobacterium moorei
Solobacterium sp. S4-A19
s__Lancefieldella_parvula|t__SGB964
s__Lancefieldella_parvula|t__SGB966
s__Lancefieldella_rimae|t__SGB985
s__Schaalia_cardiffensis|t__SGB17147
s__Schaalia_georgiae|t__SGB17149
s__Schaalia_meyeri|t__SGB17151
s__Schaalia_SGB17153|t__SGB17153
s__Schaalia_SGB17154|t__SGB17154
s__Schaalia_SGB17157|t__SGB17157
s__Schaalia_SGB17158|t__SGB17158_group
s__Schaalia_odontolytica|t__SGB17161_group
s__Schaalia_SGB17168|t__SGB17168
s__Schaalia_odontolytica|t__SGB17169
s__Schaalia_turicensis|t__SGB17195
s__Schaalia_odontolytica|t__SGB23982
s__Solobacterium_moorei|t__SGB6826
s__Solobacterium_SGB6828|t__SGB6828
s__Solobacterium_SGB6829|t__SGB6829
s__Solobacterium_SGB6833|t__SGB6833
s__Solobacterium_SGB6834|t__SGB6834
Identity [-]
B
Lp
Sm
So
88.8
97.4
87.8
ANI
