# Supplementary material for: Clinical strains isolated from early-stage colorectal cancer patients promote tumorigenesis
Source: PeerJ. 2026 Jul 14;14:e21488. doi: 10.7717/peerj.21488 (PMC13378470; doi:10.7717/peerj.21488)
Supplement: Supplemental Information 11 — (A) Small intestines. (B) Colons. All tissues were pictured in the sterilized square petri dishes of the samp dimensions (140 mm × 100 mm) [file peerj-14-21488-s011.pdf]

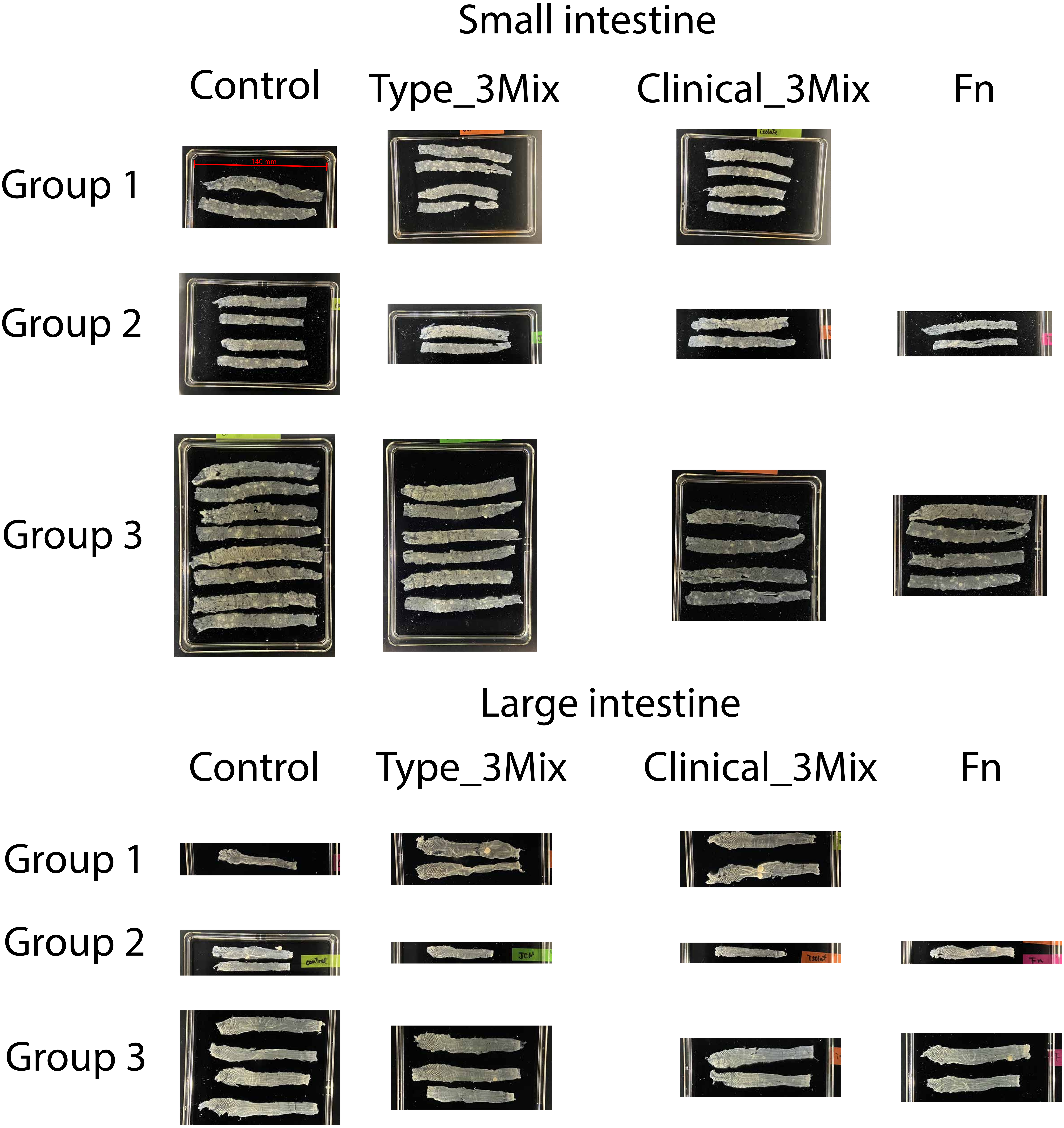
Small intestine
Control
Type_3Mix
Clinical_3Mix
Fn
Group 1
140 mm
Group 2
Group 3
Large intestine
Control
Type_3Mix
Clinical_3Mix
Fn
Group 1
Group 2
Group 3
